# Cooperative stability renders protein complex formation more robust and controllable

**DOI:** 10.1038/s41598-022-14362-z

**Published:** 2022-06-21

**Authors:** Kuan-Lun Hsu, Hsueh-Chi S. Yen, Chen-Hsiang Yeang

**Affiliations:** 1 Institute of Molecular Biology, Academia Sinica, 128 Academia Road, Section 2, Taipei, Taiwan; 2 Institute of Statistical Science, Academia Sinica, 128 Academia Road, Section 2, Taipei, Taiwan

**Keywords:** Systems biology, Genetic circuit engineering, Computational biology and bioinformatics, Protein analysis

## Abstract

Protein complexes are the fundamental units of many biological functions. Despite their many advantages, one major adverse impact of protein complexes is accumulations of unassembled subunits that may disrupt other processes or exert cytotoxic effects. Synthesis of excess subunits can be inhibited via negative feedback control or they can be degraded more efficiently than assembled subunits, with this latter being termed cooperative stability. Whereas controlled synthesis of complex subunits has been investigated extensively, how cooperative stability acts in complex formation remains largely unexplored. To fill this knowledge gap, we have built quantitative models of heteromeric complexes with or without cooperative stability and compared their behaviours in the presence of synthesis rate variations. A system displaying cooperative stability is robust against synthesis rate variations as it retains high dimer/monomer ratios across a broad range of parameter configurations. Moreover, cooperative stability can alleviate the constraint of limited supply of a given subunit and makes complex abundance more responsive to unilateral upregulation of another subunit. We also conducted an in silico experiment to comprehensively characterize and compare four types of circuits that incorporate combinations of negative feedback control and cooperative stability in terms of eight systems characteristics pertaining to optimality, robustness and controllability. Intriguingly, though individual circuits prevailed for distinct characteristics, the system with cooperative stability alone achieved the most balanced performance across all characteristics. Our study provides theoretical justification for the contribution of cooperative stability to natural biological systems and represents a guideline for designing synthetic complex formation systems with desirable characteristics.

## Introduction

Protein complexes serve as functional units in a wide range of cellular processes^1^. In stable complexes, subunits are assembled at a fixed ratio, termed stoichiometry. Maintaining subunit quantities in proportions relative to their stoichiometry is critical for both complex formation and proteostasis, as an excess of unassembled subunits may interfere with assembly processes or even have severe cytotoxic effects^2–5^. In prokaryotes, complex component genes are often located in identical operons, so co-expression is guaranteed^6,7^. In eukaryotes, subunit genes are spread across the genome, but they are often co-regulated by the same transcription factors^8,9^. Ribosome profiling has revealed that the subunit synthesis rates are approximately proportional to their stoichiometry in the logarithmic scale^10,11^. However, there is still twofold variation among the translation rates of complex subunits, implying that co-regulation of component genes is insufficient to maintain subunit stoichiometry. Moreover, other processes—such as epigenomic modification, RNA splicing, protein degradation and modification—can also influence protein quantities^12–15^. In addition, prior studies also revealed that the codon usage, mRNA degradation, and even the kinetic parameters of mRNA-binding protein can impact protein expression^16–18^. Each process is subjected to perturbations that may temporarily or continuously alter subunit quantities, hence disrupting stoichiometry. For instance, fluctuating environmental conditions such as temperature and pH, as well as varying signaling molecule concentrations, may temporarily misregulate some subunit genes^19–21^. More severely, aneuploidy and non-reciprocal recombination alters subunit gene copy numbers, dramatically and permanently increasing/decreasing subunit quantities several-fold^22,23^. Therefore, additional mechanisms beyond transcriptional and translational co-regulation are required to maintain subunit stoichiometry and minimize accumulations of unassembled subunits.

Cooperative stability is a mechanism by which a protein complex can be stabilized yet its unassembled subunits be destabilized, and it is typically achieved by degradation of unassembled subunits being much more efficient than for assembled complexes. Accordingly, protein stoichiometry is maintained because unassembled subunits are removed. Imaging and proteomics analyses have revealed such differential degradation for many protein complexes, such as fatty acid synthetase^24^, membrane protein complexes^25,26^, and ribosomes^27,28^. Two systematic studies have also suggested that cooperative stability is prevalent in multiprotein complexes^29,30^. Several theoretical studies have demonstrated that cooperative stability enhances functionality of circuits displaying oscillatory/bistable behavior and exerts a buffering effect on dosage changes^31–34^. Despite these prior studies, the advantages/disadvantages of cooperative stability in terms of various aspects of stoichiometric balance have not been explored systematically. Furthermore, in principle other mechanisms, such as negative feedback control, could maintain complex stoichiometry, yet they are rarely observed in practice^11^. How cooperative stability differs from other mechanisms enabling stoichiometric balance and why cooperative stability is favoured in natural biological systems remain unclear.

From a designer’s perspective, an ideal system should perform functions efficiently (optimality), tolerate noise or temporary fluctuations (robustness), and be readily manipulated when necessary (controllability)^35–37^. These desirable characteristics may not be achievable simultaneously, as some can be mutually contradictory. A rational process for designing an artificial biological system or justifying the presence of a natural biological system is to compare these characteristics among different systems and select the one most suitable for a specific purpose. This “design review” perspective was adopted in some early breakthrough systems biology assessments^38–40^, such as relating the abundance of feed-forward network motifs to robustness of the circuit against the shot noise of inputs. However, to our knowledge, this approach has rarely been extended to protein complex formation.

In this study, we built mathematical models of protein complex formation with or without cooperative stability and demonstrate through simulated data that cooperative stability maintains subunit stoichiometry with desirable systems characteristics. In the first part of our manuscript, we highlight two advantages of cooperative stability; a system with cooperative stability retains a high ratio of complex/monomer quantities across a broad range of parameter configurations for subunit synthesis rate and binding affinity, and complex abundance in such systems is controllable both by upregulation and downregulation of the synthesis rate of a given subunit. In the second part, we employ the design review approach to compare two mechanisms, cooperative stability and negative feedback control, postulated as contributing to complex subunit stoichiometric balance. We constructed four circuits incorporating different combinations of these two mechanisms and evaluated their performance according to eight systems characteristics. Intriguingly, the circuit with cooperative stability alone exhibited the most balanced performance for all characteristics. Our results provide valuable insights into regulatory control of protein complex and useful guidelines for designing synthetic protein complex circuits.

## Results

### An investigation of stoichiometric maintenance of protein complexes through quantitative models and simulations

To examine and compare the effects of several mechanisms contributing to stoichiometric balance, we constructed quantitative models of heteromeric protein complex formation systems (Fig. S1). In our models, two distinct protein monomers (${p}_{1}$ and ${p}_{2}$) form a heterodimer ${p}_{3}$ as a reversible chemical reaction: ${p}_{1}+{p}_{2}\rightleftharpoons {p}_{3}$ (Fig. S1A). Each protein species ${p}_{i}$ has a degradation rate constant ${\lambda }_{i}$. The net rate of change for each species (two monomeric species and one dimeric species) is represented by the rate of inflows (synthesis, disassociation/association of the monomer/dimer) minus the rate of outflows (degradation, association/disassociation of the monomer/dimer). We considered two alternative models of monomer synthesis. In the open loop system, the synthesis rates of ${p}_{1}$ and ${p}_{2}$ are given by the constants ${C}_{1}$ and ${C}_{2}$, respectively. In the negative feedback system, the concentrations of ${p}_{1}$ and ${p}_{2}$ negatively affect the transcription rates of their respective mRNAs ${m}_{1}$ and ${m}_{2}$. Likewise, we adopted two alternative models of protein degradation. In the system lacking cooperative stability, the degradation rates ${\lambda }_{1}$, ${\lambda }_{2}$, and ${\lambda }_{3}$ are all identical. In the system with cooperative stability, the degradation rate ${\lambda }_{3}$ of the dimer is less than that of the monomers ${\lambda }_{1}$ and ${\lambda }_{2}$. The abundance of each protein species is at steady state and all parameter values used in our analysis are within established physiological ranges (Table 1), mainly based on high-throughput experimental data and published databases (Fig. S2), as detailed in the Materials and Methods.

**Table 1:** Physiological ranges of model parameters.

Parameter	Description	Physiological range	Median	References
K_a_	Protein–protein association constant	0.001 ~ 1 (nM^−1^)	0.05	^41,60,61^
λ	Protein degradation rate constant	0.017 ~ 0.693 (h^−1^)	0.027	^62^
C	Protein synthesis rate	1 ~ 1000 (nM/h)	~ 100	^42^
λ_m_	mRNA degradation rate constant	0.017 ~ 2.687 (h^−1^)	0.085	^63^
K	Dissociation constant of repressor-promoter	0.001 ~ 1 (nM^−1^)		^64^
n	Cooperativity of promoter activity	1 ~ 2		^65^

For the heteromeric trimer, the system contains three distinct subunits ${p}_{1}$, ${p}_{2}$ and ${p}_{3}$. We assumed that assembly of the trimer comprises two steps (Fig. S1B), with ${p}_{1}$ and ${p}_{2}$ first forming an intermediate subcomplex ${p}_{12}$ (${p}_{1}+{p}_{2}\rightleftharpoons {p}_{12}$), and then ${p}_{12}$ binding to ${p}_{3}$ to form the trimer ${p}_{123}$ (${p}_{12}+{p}_{3}\rightleftharpoons {p}_{123}$).

### Cooperative stability maintains subunit stoichiometry across a broad range of parameter configurations

A critical property of protein complex formation systems is their ability to maintain the stoichiometric balance of individual subunits. Ideally, the quantities of subunits (monomers) are proportional to their stoichiometric coefficients in the protein complex. Moreover, all subunit molecules should be amalgamated into protein complexes so that no free monomers are left over. In practice, free monomers are rarely depleted given that the protein synthesis rates of subunits may not always adhere to their stoichiometries since genetic or environmental variations may result in continuous or temporary over- or under-expression of certain complex members.

**Figure 1:**
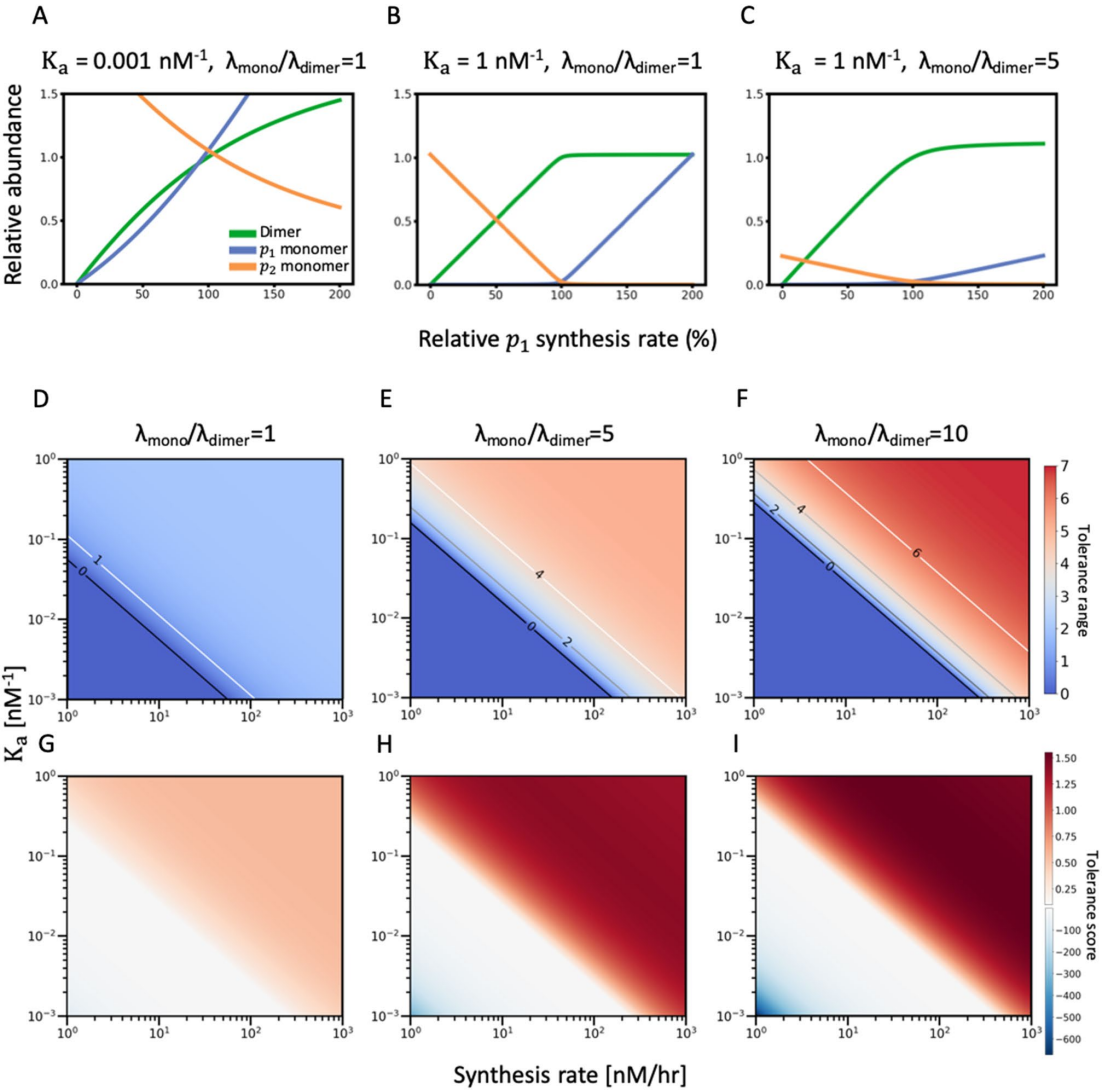
Cooperative stability allows protein complex formation systems to overcome systematic constraints on stoichiometric balance arising from synthesis rate variations. (**A**–**C**) For heterodimers, the abundance of each protein species—dimer ${p}_{3}$ (green), monomer ${p}_{1}$ (blue) and monomer ${p}_{2}$ (orange)—in response to synthesis rate variations is plotted. Protein abundance is normalized to dimer ${p}_{3}$ abundance (${C}_{1}$ = ${C}_{2}$). The ${p}_{2}$ synthesis rate ${C}_{2}$ is fixed at 50 nM/h and the ${p}_{1}$ synthesis rate ${C}_{1}$ varies from 0 to 100 nM/h (0–200% relative to the ${p}_{2}$ synthesis rate ${C}_{2}$). The response curves of each protein species in the absence of cooperative stability (${\lambda }_{monomer}{/\lambda }_{dimer}=1$) are plotted when the binding affinity is either (**A**) low (${K}_{a}=0.001 {nM}^{-1}$) or (**B**) high (${K}_{a}=1 {nM}^{-1}$). Response curves of each protein species subjected to cooperative stability (${\lambda }_{monomer}{/\lambda }_{dimer}=5$) and with high binding affinity (${K}_{a}=1 {nM}^{-1}$) are shown in (**C**). (**D**–**F**). The heatmap of the tolerance range with parameter configurations in the physiological range. The x-axis is the reference protein synthesis rate (${C}_{2}$) from 1 to 1000 nM/h and the y-axis is the association constant ${K}_{a}$ from 0.001 to 1 nM^−1^, both axes are at log scale. (**G**–**I**) The heatmap of the tolerance score within 0 to two-fold synthesis rate variation range in the same parameter space of ${K}_{a}$ and ${C}_{2}$ as (**D**–**F**). For heterodimers without cooperative stability, the heatmap is plotted in (**D** and **G**). For heterodimer with cooperative stability, the heatmaps are plotted for different extent of differential degradation rate using ${\lambda }_{monomer}{/\lambda }_{dimer}=5$ (**E** and **H**) and ${\lambda }_{monomer}{/\lambda }_{dimer}=10$ (**F** and **I**).

To determine how the stoichiometry of a protein complex responds to imbalanced synthesis rates of its constituent monomers, we fixed the synthesis rate of one subunit ${p}_{2}$ and varied the synthesis rate of the other subunit ${p}_{1}$, before examining the normalized abundance of all three protein species (relative to the quantities when the synthesis rates of ${p}_{1}$ and ${p}_{2}$ are equal) in simulated data (Fig. 1A-C). In the absence of cooperative stability (${\lambda }_{monomer}{=\lambda }_{dimer}$) and when the binding affinity between protein species is low (${K}_{a}=0.001 {\mathrm{nM}}^{-1}$), relative abundance of the dimer never exceeds that of the monomer, with the synthesis rate of ${p}_{1}$ ranging from 0 to 200% that of ${p}_{2}$ (Fig. 1A). This outcome is anticipated considering that the protein concentration ratio at equilibrium $\frac{[{p}_{3}]}{\left[{p}_{1}\right][{p}_{2}]}\equiv {K}_{a}$ is low. In this case, the amounts of unassembled monomers surpass that of complexes even if the synthesis rates of both constituent subunits are stoichiometrically balanced (1:1 in heterodimer). Increasing the binding affinity ${K}_{a}$ to $1 {\mathrm{nM}}^{-1}$ elevates the equilibrium concentration of ${p}_{3}$ and lowers those of ${p}_{1}$ and ${p}_{2}$, thereby rendering dimers more abundant than monomers across a broad range of ${p}_{1}$ synthesis rates (Fig. 1B). However, the influence of ${K}_{a}$ on the dimer/monomer ratio is limited. We introduce two quantitative measures for the robustness of stoichiometry maintenance against parameter variations. First, we highlight the range of ${p}_{1}$ synthesis rates where dimer abundance surpasses monomer abundance (shaded areas in Fig. S3A), and define a “Tolerance range” as the log2 ratio of the upper and lower limits of this range. As expected, increasing ${K}_{a}$ expands the upper range of dimer/monomer ratios, thereby increasing the tolerance range (Fig. S3A). However, the tolerance range cannot exceed 2.0 (the ${p}_{1}$ synthesis rate ranges from 50 to 200% of the reference) even when the binding affinity of streptavidin–biotin is considered (${K}_{a}=10000 {\mathrm{nM}}^{-1}$), known to be the strongest non-covalent interaction in nature.

The tolerance range upon varying the ${p}_{1}$ synthesis rate can also be expanded by increasing the reference ${p}_{2}$ synthesis rate ${C}_{2}$ (Fig. S3B), as increasing ${C}_{2}$ bolsters the limited ${p}_{2}$ supply and hence shifts the equilibrium state toward the right-hand-side of the chemical reaction (greater ${p}_{3}$ concentration). However, ${C}_{2}$ exerts the same limitation on the dimer/monomer ratio as ${K}_{a}$ (Fig. S3A), because enhancing ${C}_{2}$ from 10 to 1000 only elevates the tolerance range from 1.77 to 1.997. We mathematically deduce this observed constraint (tolerance range < 2) in Supplementary File S1.

When cooperative stability (${\lambda }_{monomer}{>\lambda }_{dimer}$) is introduced to the system, the amounts of unassembled subunits caused by synthesis rate variation are significantly reduced (Fig. 1C). Moreover, the tolerance range can be elevated to 5 when the ${\lambda }_{monomer}{/\lambda }_{dimer}$ ratio is 5 or to 7 when it is 10 (Fig. S4), indicating that cooperative stability may overcome the limitations of ${K}_{a}$ and ${C}_{2}$ to maintain higher dimer/monomer ratios across a broader range of ${p}_{1}$ synthesis rates. Increasing the ${\lambda }_{monomer}{/\lambda }_{dimer}$ ratio induces a similar yet more pronounced effect as achieved by increasing ${K}_{a}$, as cooperative stability lowers the equilibrium concentrations of monomers (due to their higher degradation rates) and hence elevates the $\frac{[{p}_{3}]}{\left[{p}_{1}\right][{p}_{2}]}$ ratio. Although cooperative stability facilitates maintenance of complex subunit stoichiometry, it might be detrimental to complex formation if the monomers are degraded too efficiently, so that the probability of monomers encountering each other is diminished. Indeed, there is a maximum extent to which cooperative stability can enhance the tolerance range and the value also depends on ${K}_{a}$ and ${C}_{2}$ (Fig. S5, see also Supplementary File S1). The ${\lambda }_{monomer}{/\lambda }_{dimer}$ ratio does not affect the tolerance range for small ${K}_{a}$ and ${C}_{2}$ values (Fig. S5A-B) because complex formation is presumably difficult under such conditions. When ${K}_{a}$ and ${C}_{2}$ exceed threshold values, the tolerance range peaks at an intermediate ${\lambda }_{monomer}{/\lambda }_{dimer}$ value and is consistently reduced for both high and low ${\lambda }_{monomer}{/\lambda }_{dimer}$ values (Fig. S5C).

The tolerance range concerns only the protein synthesis rate range above a particular threshold (dimer/monomer ratio $\ge$ 1.0), but does not consider the quantitative difference between the dimer and monomer abundances, and ignores the parameter regimes where the monomer abundance exceeds the dimer abundance. To overcome these limitations, we introduce the second measure termed “Tolerance score” as the difference of the areas under the dimer curve (green curve in Fig. 1A–C) and the supremum of the monomer curve (max of blue and orange curves in Fig. 1A–C) over the twofold synthesis rate variations (0%–200% of the reference synthesis rate)^11^. The tolerance score concerns the entire range of synthesis rates and quantitative difference between dimer and monomer abundances, yet it also intermingles the information about the synthesis rates range and dimer/monomer abundance difference. In contrast, the tolerance range focuses on the synthesis rate range. Therefore, we use both tolerances ranges and tolerance scores to quantify stoichiometry robustness and visualize them against a wide range of two parameter values (${p}_{2}$ synthesis rates and association constants) for three scenarios of cooperative stability (Fig. 1D–I).

For systems lacking cooperative stability (${\lambda }_{monomer}{/\lambda }_{dimer}=1$), the tolerance range and tolerance score do not surpass 2 and 0.5 in the range of three orders of magnitude for ${K}_{a}$ and ${C}_{2}$ (Fig. 1D and G). When ${\lambda }_{monomer}{/\lambda }_{dimer}=5$, both tolerance range and tolerance score significantly increase and exceed 4 and 1 in about one half of the parameter configuration space (Fig. 1E, H). Furthermore, when ${\lambda }_{monomer}{/\lambda }_{dimer}=10$, many more parameter configurations yield a tolerance range > 6 and a slightly increased tolerance score (Fig. 1F, H). Intriguingly, although cooperative stability substantially raises both tolerance measures in about one half of the parameter configuration space examined, it also moderately expands the space of undesirable parameter configurations (where the tolerance range $=0$, or the tolerance score $\le 0$; blue regimes in Fig. 1D–F and white regimes in Fig. 1G–I). These properties indicate that the benefits of cooperative stability are manifested only when the synthesis rates and binding affinities are sufficiently high, since differential degradation takes effect only when enough proteins are synthesized and formed. Also, the tolerance scores are excessively negative with small synthesis rates and binding affinities, since under these conditions dimers are hardly formed and hence the relatively abundance of monomers far exceeds that of dimers.

Given the considerable proportion of the undesirable parameter configuration space, we have to justify the benefits of cooperative stability by showing that most physiologically relevant protein complexes fall in the desirable regimes of the parameter configuration space (red areas in Fig. 1D–I). We collected 11 protein complexes both annotated in PDBbind (a protein–protein interaction database)^41^ and examined by a systematic protein synthesis rate study^42^, and marked their monomer synthesis rates and association constants in the parameter configuration space (light blue dots in Fig. S6). Intriguingly, 8 of 11 protein complexes fall in the regimes with positive tolerance scores, and 6 of 11 protein complexes are in the high tolerance areas (tolerance scores $\ge 1.25$). To sum up, these results demonstrate that cooperative stability is an effective mechanism for maintaining subunit stoichiometry and its effect is manifested in a large portion of the physiologically relevant parameter space.

### Protein complex formation systems with or without cooperative stability display asymmetric levels of controllability with respect to the direction of synthesis rate variation

**Figure 2:**
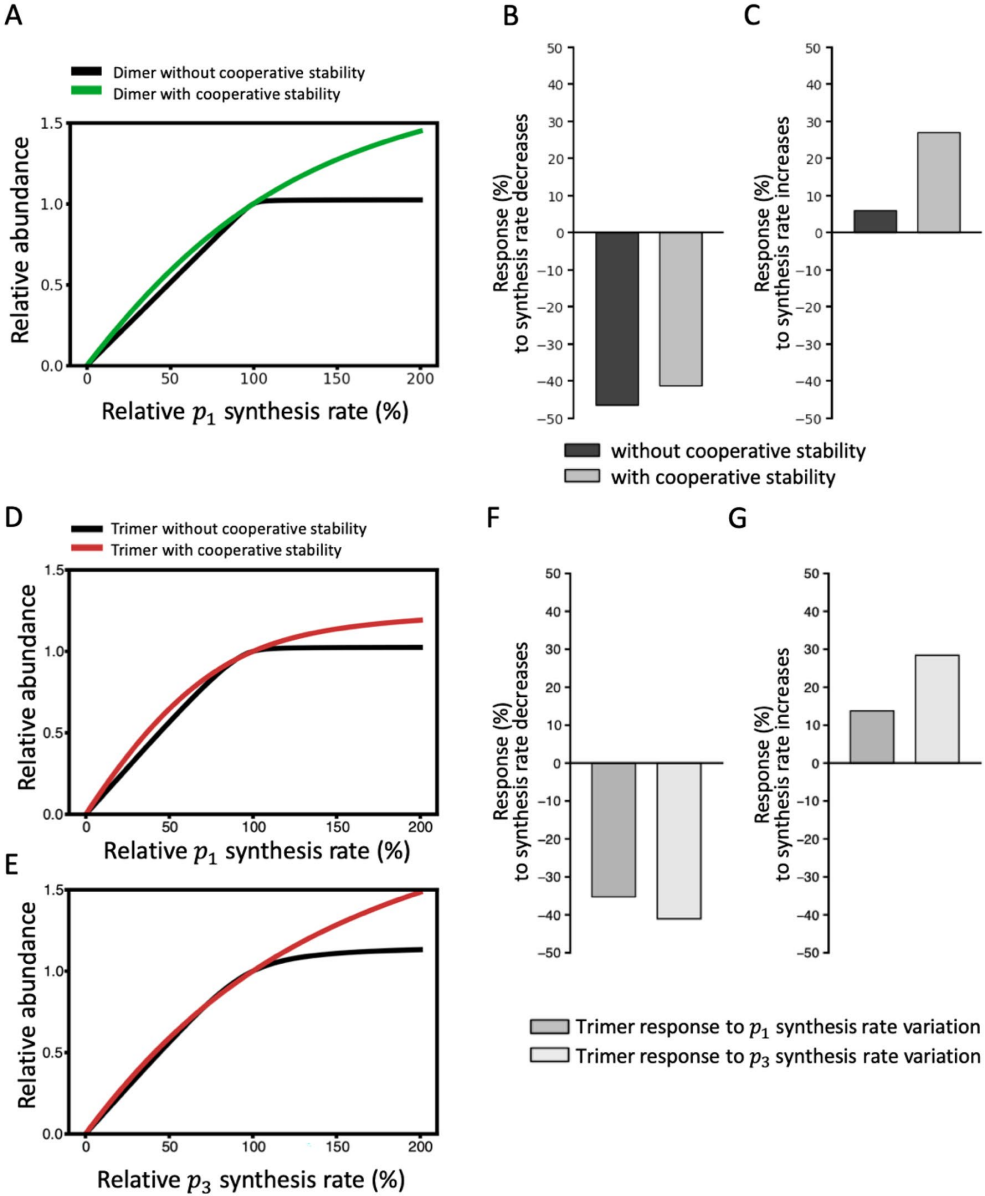
Protein complex formation systems with or without cooperative stability display asymmetric levels of controllability with respect to the directions of synthesis rate variation. (**A**) Dimer abundance in response to synthesis rate variations without cooperative stability (black) or with cooperative stability (green). Dimer abundance is normalized to dimer abundance when the ${p}_{1}$ and ${p}_{2}$ synthesis rates are the same. The ${p}_{2}$ synthesis rate is fixed at 100 nM/h and the ${p}_{1}$ synthesis rate varies from 0 to 200 nM/h. (**B**) Quantification of the dimer abundance response without (black) or with (gray) cooperative stability when the synthesis rate is reduced by 50% (from 100 to 50 nM/h). (**C**) Quantification of the dimer abundance response without (black) or with (gray) cooperative stability when the synthesis rate is increased by 50% (from 100 to 150 nM/h). (**D**, **E**) Trimer abundance response curves without (black) or with (red) cooperative stability arising from synthesis rate variation of ${p}_{1}$ (**D**) and ${p}_{3}$ (**E**). (**F**, **G**) Quantification of the trimer abundance response to ${p}_{1}$ (gray) and to ${p}_{3}$ (light gray) synthesis rate variations, i.e., a 50% decrease (**F**) and a 150% increase (**G**). The association constant ${K}_{a}$ is 0.05 nM^−1^ in all plots.

Apart from dimer/monomer ratios, another characteristic of complex formation systems is controllability, reflecting how sensitive complex concentrations are to unilateral upregulation or downregulation of one subunit. Accordingly, we examined how a system responds in terms of dimer abundance when the ${p}_{1}$ synthesis rate varies relative to a fixed ${p}_{2}$ synthesis rate (Fig. 2A). If the system lacks cooperative stability, the dimer abundance curve bends sharply at the reference synthesis rate (100%), illustrating how dimer quantities are reduced with decreasing ${p}_{1}$ synthesis rates but remain almost invariant with increasing ${p}_{1}$ synthesis rates. In contrast, when cooperative stability is a feature of the system, the dimer abundance curve bends only very slightly, reflecting more symmetric responses with respect to ${p}_{1}$ synthesis rate variations in both directions. Thus, dimer quantities generally slowly decrease with decreasing ${p}_{1}$ synthesis rates and slowly increase with increasing ${p}_{1}$ synthesis rates. Quantitatively, the declines in dimer abundance in response to a 50% decrease in ${p}_{1}$ synthesis rates are very similar between systems with or without cooperative stability (Fig. 2B), but the elevations in dimer abundance in response to a 50% increase in ${p}_{1}$ synthesis rates are considerably higher for a system with cooperative stability compared to one without it (Fig. 2C).

Although we have generated Fig. 2A–C from a specific parameter configuration, the effect of cooperative stability on symmetric responses to bidirectional changes in subunit synthesis rates is robust across a wide range of parameter configurations. In Fig. S7A-B, we illustrate the system response in terms of dimer abundance to a 150% increase in the ${p}_{1}$ synthesis rate while varying ${C}_{2}$ and ${K}_{a}$ up to a 1000-fold, and with or without cooperative stability. Fig. S8A-B displays the system response to a 50% ${p}_{1}$ synthesis rate decrease under the same conditions. The system in which ${\lambda }_{monomer}{/\lambda }_{dimer}$=1 exhibits insensitivity in terms of responses to an increase in the ${p}_{1}$ synthesis rate (upward response $\le$ 120%) for most of the parameter configurations (Fig. S7A), whereas the system with ${\lambda }_{monomer}{/\lambda }_{dimer}$=5 displays greater sensitivity in terms of upward responses ($\ge$ 130%) for more than half of the parameter configurations (Fig. S7B). In contrast, the systems with ${\lambda }_{monomer}{/\lambda }_{dimer}$=1 and ${\lambda }_{monomer}{/\lambda }_{dimer}$=10 have narrow and similar downward responses or similar ranges of parameter configurations (Fig. S8A-B). A system with cooperative stability has a much wider regime of parameter configurations for sensitive upward responses (black band in Fig. S7E) than a system without cooperative stability (Fig. S7D), yet the regimes for sensitive downward responses (Fig. S8C-D) are comparable between systems with or without cooperative stability.

To determine if the effect of cooperative stability on heterodimer responsiveness also applies to higher-order protein complexes, we examined responses in terms of trimer abundance upon varying the synthesis rate of one subunit while fixing the synthesis rates of the remaining two subunits (Fig. 2D, E). In the absence of cooperative stability, the trimer abundance curve also bends sharply at the reference synthesis rate (100%) and trimer quantities barely change, which is a similar outcome to our observations for dimer shown in Fig. 2A. In contrast, in a system with cooperative stability, trimer quantities are elevated upon increasing the synthesis rate of ${p}_{1}$ or ${p}_{3}$. Thus, protein complexes subjected to cooperative stability are sensitive to synthesis rate variations in either direction (i.e., upward or downward). Intriguingly, the trimer response curves arising from ${p}_{1}$ and ${p}_{3}$ synthesis rate variations are different to those of dimer. By varying the ${p}_{3}$ synthesis rate, the system responds more sensitively in terms of trimer abundance to both upward or downward rate variations (50% and 150%) compared to when the ${p}_{1}$ synthesis rate is varied (Fig. 2F, G). That scenario is intuitive since the effect of monomers at an early stage of complex formation (see ${p}_{1}$ in Fig. S1B) attenuates with a larger extent compared to the effect of monomers at intermediate steps. Taken together, these results indicate that cooperative stability allows protein complex formation systems to be more controllable upon unilateral up/downregulation of a given subunit. Moreover, the controllability of higher-order protein complexes is associated with the assembly order of the subunits responsible for regulating complex abundance.

### Stoichiometric regulation through cooperative stability displays balanced strength in terms of optimality, controllability, and robustness

**Figure 3:**
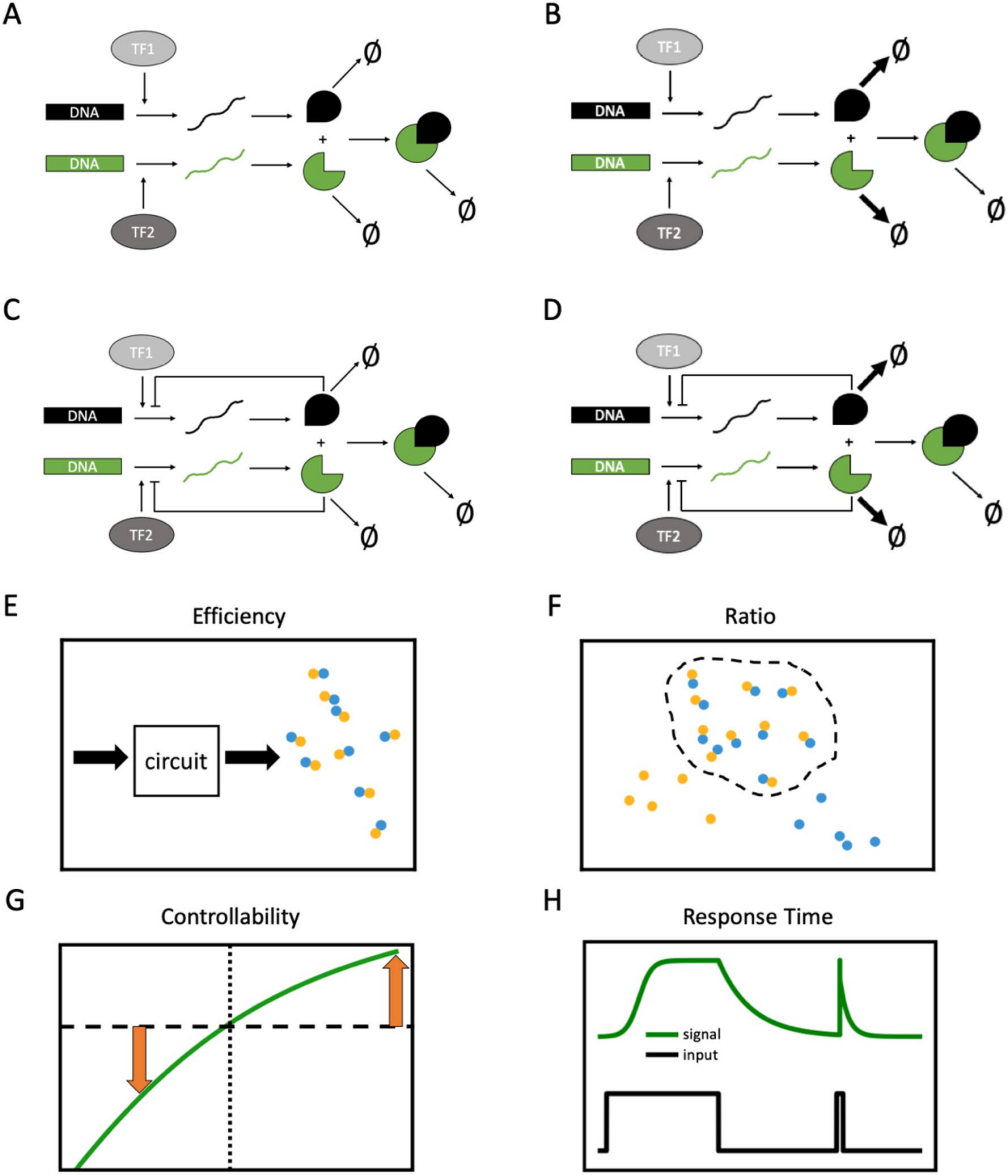
Schematics of our model circuits and characteristics of a protein complex formation system. (**A**–**D**) Diagrams of the four assessed circuits. (**A**) Circuit 1 (open loop without cooperative stability). (**B**) Circuit 2 (open loop with cooperative stability). (**C**) Circuit 3 (negative feedback loops without cooperative stability). (**D**) Circuit 4 (negative feedback loops with cooperative stability). (**E**–**H**) Schematics of the characteristics of a protein complex formation system. (**E**–**G**) Equilibrium properties, i.e. (**E**) efficiency, (**F**) dimer ratio, and (**G**) controllability, of a system at steady-state. (**H**) Dynamic behaviors of the system, i.e., response time to upward regulation, response time to downward regulation, and recovery time after a shot noise.

**Figure 4:**
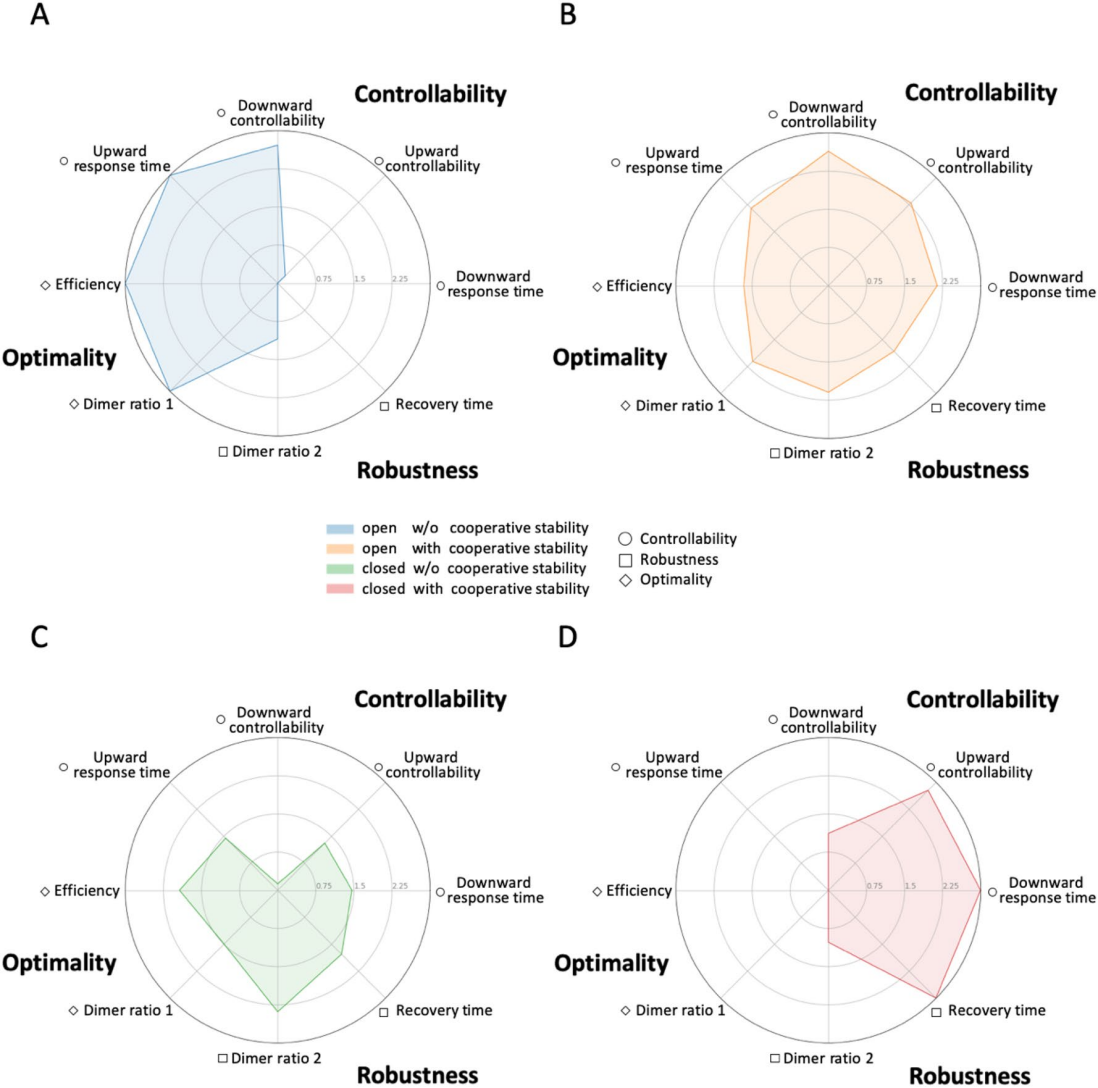
Circuit performance according to multiple design characteristics. Radar charts representing the relative performance of each circuit according to eight characteristics across the parameter space. The score is the result of multiple comparison between a given circuit and the other three circuits. The eight characteristics are grouped into three categories: (1) Optimality [Efficiency and Dimer ratio 1 (stoichiometrically balanced)], denoted by diamonds; (2) Robustness [(Dimer ratio 2 (stoichiometrically imbalanced)) and Recovery time]], denoted by squares; and (3) Controllability [Upward/Downward controllability and Upward/Downward response time], denoted by circles. (**A**) Circuit 1 (open loop without cooperative stability) (blue). (**B**) Circuit 2 (open loop with cooperative stability) (orange). (**C**) Circuit 3 (negative feedback loops without cooperative stability) (green). (**D**) Circuit 4 (negative feedback loops with cooperative stability) (red).

We have demonstrated that a protein complex with cooperative stability maintains subunit stoichiometry across a broad range of parameter configurations and induces sensitive responses of dimer abundance to unilateral changes in the synthesis rate (upwards or downwards) of any given monomer. Both these benefits stem from the system’s capability to efficiently remove unassembled monomers. That ability could also be achieved by negative feedback control of monomer synthesis. We wondered which mechanism is more prevalent in nature and which is more appropriate for designing synthetic protein complex circuits. To address those questions, we conducted in silico experiments to generate simulated data from circuits incorporating these mechanisms and then compared their performance according to several aspects of circuit design. The process for reporting circuit performance is illustrated in Fig. 3. We considered four circuits encompassing possible combinations of the two mechanisms. Circuit 1 lacks both cooperative stability and negative feedback control of monomer synthesis (Fig. 3A). Circuit 2 has cooperative stability, but no negative feedback control (Fig. 3B). Circuit 3 has two negative feedback loops whereby each protein monomer directly inhibits transcription of its own mRNA transcription, but it lacks cooperative stability (Fig. 3C). Circuit 4 displays cooperative stability and also possesses the two negative feedback loops (Fig. 3D). Simulated data generated from each circuit were assessed according to eight systems characteristics, five of which are steady-state properties: (1) efficiency—the number of dimeric proteins produced by each mRNA molecule (Fig. 3E); (2) dimer ratio 1—the ratio of dimer to total protein quantities when the ${p}_{1}$ and ${p}_{2}$ synthesis rates are identical (stoichiometrically balanced) (Fig. 3F); (3) dimer ratio 2—the ratio of dimer to total protein quantities when the ${p}_{1}$ synthesis rate is two-fold that of the ${p}_{2}$ synthesis rate (stoichiometrically imbalanced) (Fig. 3F); (4) upward controllability—the increase in dimer quantities when the ${p}_{1}$ synthesis rate is increased two-fold (Fig. 3G); and (5) downward controllability—the decrease in dimer quantities when the ${p}_{1}$ synthesis rate is decreased two-fold (Fig. 3G). The three remaining systems characteristics are kinetic properties: (6) upward response time—the duration for dimer quantities to reach a threshold after both monomer synthesis rates have risen abruptly to a high value (Fig. 3H); (7) downward response time—the duration for dimer quantities to fall below a threshold after both monomer synthesis rates have abruptly dropped to a low value (Fig. 3H); and (8) recovery time—the duration for monomer quantities to return to the steady-state value after an impulse perturbation (Fig. 3H). These eight characteristics capture three general aspects of biological circuit design. First, optimality characteristics (efficiency, dimer ratio 1, upward response time) quantify behaviors under conditions of stoichiometric balance or optimal conditions. Second, robustness characteristics (dimer ratio 2, recovery time) quantify stoichiometric maintenance and recovery from perturbations. Third, controllability characteristics (upward/downward controllability, upward/downward response time) quantify responsiveness to upward/downward synthesis rate variations.

We compared the performance of our four protein complex circuits based on these eight characteristics according to the following procedures. Simulated data for the four circuit models with 10,000 parameter configurations were generated within the physiologically relevant ranges reported in Table 1. For each characteristic, we measured the performance of a circuit in terms of the probability that it is superior to other circuits over the sampled parameter configurations. However, since these circuits do not possess the same set and number of parameters, it is more fair to compare pairs of circuits rather than all circuits at once. Therefore, we propose an indirect approach to measure circuit performance through pairwise comparisons. In brief, for each pair of circuits we calculated the probability that one circuit outperforms another over 10,000 parameter configurations. The probability of the ranking order of four circuits in a permutation is proportional to the product of probabilities of all compatible pairwise orders. The probability of the ranking of one circuit is marginalized from the probabilities of the ranking orders of four circuits. Finally, the performance index of one circuit is the area under the cumulative mass function of its probability of the ranking. A detailed protocol is described in the Materials and Methods.

In Fig. 4, we illustrate by means of radar charts the relative performance of the four circuits for all eight characteristics at once by which we could unbiasedly compare the overall performance profiling of each circuit. Circuit 1 (open loop without cooperative stability, Fig. 4A) achieved the highest performance in all optimality indices, but the lowest performance in all robustness indices and for some controllability indices (upward controllability, downward response time). Circuit 2 (open loop with cooperative stability, Fig. 4B) displayed a very balanced performance of all characteristics. Although its optimality indices are inferior to those of Circuit 1, they are better than for the other two circuits. Moreover, its robustness indices are considerably superior to those of circuit 1 and they are comparable to those of the other two circuits. Its controllability indices are generally superior to those of circuits 1 and 3 and they are comparable to those of Circuit 4. Circuit 3 (negative feedback loops without cooperative stability, Fig. 4C) presented the best performance for one robustness index (dimer ratio 2), but displayed slightly inferior performance to Circuit 2 for optimality indices and overall poor performance for controllability indices. Circuit 4 (negative feedback loops with cooperative stability, Fig. 4D) exhibited superior performance to other circuits for one robustness index (recovery time) and two controllability indices (upward controllability and downward response time), yet its performance was poor for all remaining indices.

We deduce several important attributes from our comparative assessment of the four circuits. No circuit universally outperformed the other circuits for all characteristics, with individual circuits displaying distinct strengths and weaknesses. Generally, the circuit lacking mechanisms to maintain subunit stoichiometry (Circuit 1) achieves the best performance with respect to optimality when stoichiometry is already in balance. However, that circuit is vulnerable if subunit stoichiometry is perturbed by genetic or environmental variations. Cooperative stability significantly improves circuit robustness (compare circuits 1 and 2). In addition, cooperative stability enhances controllability of an open loop circuit (compare circuits 1 and 2) and the dual negative-feedback loop circuit (compare circuits 3 and 4). This enhanced controllability exerted by cooperative stability supports our findings presented in Fig. 2, indicating that cooperative stability enables a protein complex to be more controllable independently of negative feedback control. Negative feedback-regulated synthesis also improves circuit robustness in the absence of cooperative stability (compare circuits 1 and 3), yet Circuit 4 that was equipped with both mechanisms only achieved best performance for three indices, so dual regulatory mechanisms do not additively increase circuit performance. This comparative analysis reveals that cooperative stability and negative feedback exert distinct effects. Hence, although they both maintain subunit stoichiometry, they endow different characteristics on protein complex formation systems.

## Discussion

Here, we have investigated the role of cooperative stability in the formation and regulation of protein complexes by means of quantitative models and simulated data. We have identified two properties markedly distinguishing systems with or without cooperative stability. First, a protein complex subjected to cooperative stability maintains a higher complex/monomer abundance ratio over a wide range of parameter configurations. Second, cooperative stability facilitates sensitive responses in terms of complex abundance to either decreasing or increasing rates of monomer synthesis. We further expanded the scope of our study by generating four circuits of protein complex formation that incorporated combinations of two mechanisms for regulating subunit stoichiometry, i.e., cooperative stability and negative feedback control, and then comparing their performance according to eight characteristics pertaining to system optimality, controllability, and robustness. Although a number of previous studies have investigated the benefits of cooperative stability in protein complex formation, the two properties of robust stoichiometry maintenance and bidirectional controllability reported herein remain less well explored. Moreover, despite synthetic biological circuits being the subject of numerous studies, their primary focus tends to be either on general design principles^43,44^ or implementation of systems with specific functions^45,46^, and very few have determined desirable characteristics or compared circuits in terms of those characteristics. Hence, our approach may be generalized to other systems beyond protein complexes.

The robustness and controllability endowed by cooperative stability provide strong evidence for the selective advantage of this regulatory mechanism in enabling protein complexes to respond to environmental stimulations such as stress responses^47,48^. Through cooperative stability, a protein complex can swiftly respond to a wide range of system variations induced by environmental/cellular stimulations and without the need for reprogramming of the entire regulatory circuit or adversely impacting proteostasis. Accordingly, the system can evolve further independent regulatory circuits that are amenable to modularization. Moreover, the incoherence among how different subunits are synthesized creates additional degrees of freedom to develop complex regulatory logics.

Our systematic characterization of four theoretical protein complex circuits incorporating combinations of two regulatory mechanisms potentially contributing to stoichiometric balance (Fig. 4) supports that diverse mechanisms may act in natural or synthetic systems to cope with various contexts and requirements. Each of the four putative circuits displayed strengths and weaknesses for distinct design aspects, and none of the circuits was universally superior or inferior for all characteristics. Thus, cooperative stability, negative feedback control, and an absence of any mechanism of stoichiometric balance each endow distinct advantages in terms of circuit controllability, robustness and optimality. Cooperative stability enables a protein complex to respond swiftly to unilateral changes of inputs (Fig. 2), thereby rendering the system controllable. Negative feedback loops are well known in control theory as system stabilizers^49^. A circuit lacking mechanisms of stoichiometric balance is superior under the optimality condition since it does not incur the unnecessary cost (excessive degradation or inhibition of monomers) borne by circuits that do have them. For Circuit 1 (without any mechanism of stoichiometric balance) and Circuit 3 (solely having negative feedback control), their respective strengths in optimality or robustness are accompanied by weaknesses in the other two traits. Consequently, Circuit 1 is likely most appropriate for abundant housekeeping protein complexes that already have fine-tuned regulatory circuits mediating their components to maintain subunit stoichiometry, do not need to respond to environmental/internal changes, and tolerate minor fluctuations and noise. In contrast, Circuit 3 is likely best for protein complexes requiring stable production or rigorously controlled stoichiometric balance, such as the gp6–gp7 complex of HK97 phage given that imbalance of its two constituent subunits interferes with complex assembly^50^.

Cooperative stability endows both robustness (Fig. 1) and controllability (Fig. 2) on protein complex formation, with the circuit incorporating cooperative stability alone (Circuit 2) achieving the most balanced performance across all eight assessed characteristics (Fig. 3). Therefore, Circuit 2 meets the highest number of design criteria and likely operates in most natural protein complexes. This speculation is supported by two systematic studies showing that many protein complex subunits exhibit non-exponential degradation^30^ and subunits overexpressed or encoded by extra chromosomes are post-translationally buffered via protein degradation^22,29,30^. Furthermore, systematic quantification of protein synthesis rates in aneuploid cells has shown that most protein complex subunits are not subjected to feedback regulation^11^.

Curiously, the benefits of the two mechanisms of stoichiometric balance are not additive. Though our circuit encompassing both cooperative stability and negative feedback control (Circuit 4) achieved the best performance for three indices pertaining to controllability and robustness, it displayed the worst performance for almost all of the remaining indices. Consequently, combining both mechanisms is clearly more costly than either alone under optimal conditions, significantly impairing the performance of optimality indices. Moreover, performance of some of the robustness and controllability indicators is modest for Circuits 2 and 3, but quite poor for Circuit 4. Protein complex formation systems appear to operate best when the protein complexes need to recover quickly from transient perturbations and respond to persistent changes of inputs, but the extra resources consumed to buffer against such challenges are not critical, as reported for the hypoxia-Inducible factor heterodimer^51^.

It is curious that the efficiency ($\frac{{p}_{3}}{{m}_{1}}$ at steady state) performance of Circuit 3 is considerably inferior to Circuit 1 since negative feedback loops presumably affect only transcription rates but not protein synthesis or degradation rates. Explanation for this counter-intuitive behavior requires delving into the model equations. Feedback loops directly regulate the mRNA synthesis rates through free monomer concentrations (Eqs. 27–28), but this additional regulation alters both ${m}_{1}$ and ${p}_{3}$ values at steady state. In the closed loop circuit, both ${m}_{1}$ and ${p}_{3}$ values decrease compared to the open loop circuit. Yet ${p}_{3}$ reduces more than ${m}_{1}$, and hence the efficiency in circuit 3 is lower than circuit 1. As an approximation, we assume the dimer degradation rate ${\lambda }_{3}$ is negligible in Eq. (26), hence ${k}_{on}{p}_{1}{p}_{2}-{k}_{off}{p}_{3}=0$. In Circuit 1, steady state ${m}_{1}$ and ${m}_{2}$ are determined by model parameters independent of circuits, hence can be treated as constants ${c}_{1}$ and ${c}_{2}$. The steady state ${p}_{1}$ and ${p}_{2}$ from Eqs. (24–25) become ${p}_{1}=\frac{{K}_{t1}{c}_{1}}{{\lambda }_{1}}, {p}_{2}=\frac{{K}_{t2}{c}_{2}}{{\lambda }_{2}}$, and from simplified Eq. (26) ${p}_{3}={K}_{a}{p}_{1}{p}_{2}=\frac{{K}_{a}{K}_{t1}{K}_{t2}{c}_{1}{c}_{2}}{{\lambda }_{1}{\lambda }_{2}}$, and $\frac{{p}_{3}}{{m}_{1}}=\frac{{K}_{a}{K}_{t1}{K}_{t2}{c}_{2}}{{\lambda }_{1}{\lambda }_{2}}$. In Circuit 3, according to Eqs. (27–28) the steady state ${m}_{1}$ and ${m}_{2}$ are approximately ${m}_{1}={c}_{1}\frac{K}{K+{p}_{1}}, {m}_{2}={c}_{2}\frac{K}{K+{p}_{2}}$, and ${p}_{1}=\frac{{K}_{t1}{c}_{1}}{{\lambda }_{1}}\frac{K}{K+{p}_{1}}, {p}_{2}=\frac{{K}_{t2}{c}_{2}}{{\lambda }_{2}}\frac{K}{K+{p}_{2}}.$ Thus ${p}_{1}$ and ${p}_{2}$ are solutions of quadratic equations. Approximately, the dimer quantity in Circuit 3 scales with a square root ${p}_{3}={K}_{a}K\sqrt{\frac{{K}_{t1}{K}_{t2}{c}_{1}{c}_{2}}{{\lambda }_{1}{\lambda }_{2}}}$, while the mRNA quantity in Circuit 3 scale with a hyperbola function with a much slower rate (${{m}_{1}=c}_{1}\frac{K}{K+{p}_{1}})$ since reduction of ${p}_{1}$ is buffered by constant $K$. Therefore, the ratio $\frac{{p}_{3}}{{m}_{1}}$ becomes smaller in a closed loop circuit.

The radar charts in Fig. 4 give an impression that all characteristics are of the same importance. In fact, they are merely a visualization tool to concurrently display the performance of multiple characteristics in the same charts. We treat each characteristic independently from others but do not assume their relative importance. Moreover, the magnitude along each direction of the charts is obtained from the relative performance (ranking orders) of the circuits over parameter configurations, and is not directly tied to the scales of each performance index. The relative importance of these characteristics is subjective and depends on the specific tasks the circuit will achieve. In principle, one may assign different importance to each characteristic (depending on specific design goals) and rescales the axes accordingly.

Besides cooperative stability, cotranslational assembly is another important mechanism to regulate subunit stoichiometry of protein complexes. Cotranslational assembly is common in cells^52^. Furthermore, McShane et al.^30^ showed that non-exponentially degrading proteins are often subunits of complexes that assemble early, and are thus more likely to assemble cotranslationally. These lines of evidence suggest that co-translated complex subunits might tend to possess cooperative stability. On the other hand, from an evolutionary perspective a major benefit of cooperative stability is robustness against unilateral fluctuation of subunit synthesis. Since cotranslationally assembling subunits assemble together when attached to ribosomes, they might not need an extra mechanism to regulate their stoichiometry. Consequently, cooperative stability and cotranslational assembly might serve as complementary mechanisms to maintain subunit stoichiometry. It remains an open problem to resolve these two speculations as to our knowledge no proteome-wide concurrent interrogation of the two processes has yet been undertaken.

Our study represents an exploratory assessment to better understand the theoretical roles of protein degradation mechanisms in protein complex formation. To achieve that goal, we adopted a “design review” approach to compare the performance of several circuits according to diverse indices pertaining to system characteristics. Despite the significant implications we have deduced from simulated data, the study is limited in several respects, so there is considerable scope for future study. First, our model treats complex formation as a chemical reaction of free molecules, overlooking the machineries involved in facilitating subunit associations/disassociations such as scaffolds and chaperons. Second, stoichiometric balance is a crucial but not the only desirable property of protein complex formation. We have not addressed other important properties, such as delivery of protein complexes to the correct locations and their removal when no longer needed. Third, even within the scope of stoichiometric balance, other regulatory mechanisms may be active, such as provision of bait molecules to divert subunits or controlled subunit localization, which have not been considered herein. Fourth, the eight indices encompassing three design aspects certainly cannot be considered as systematically describing all of the characteristic requirements of a biological circuit, though together they do represent an adequate overview. Fifth, our model is generic and does not represent any specific protein complex. Focusing on a specific system, enriching the model with additional pertinent detail, and generating experimental data for such a system would help validate the conclusions we have derived from our simulated data and provide deeper insights into how protein complex formation is regulated. From a synthetic biology perspective, introducing cooperative stability into a protein complex system is more challenging than endowing a system with negative feedback control of mRNA synthesis since our collective knowledge of protein degradation mechanisms is much less complete than that of mRNA synthesis. A feasible method of programing protein degradation, and thus exerting cooperative stability, is to insert/delete degrons (short amino acid motifs recognized by the protein degradation machinery) into target proteins^53–56^. Efforts to overcome these limitations are warranted to provide a more holistic overview of the regulatory control of protein complex formation systems and represent promising avenues for future research.

## Methods

### Heterodimer

Three differential equations describing the dynamics of the three species in heterodimer formation system are (Fig. S1A):

1 $${\frac{d{p}_{1}}{dt}=C}_{1}+{k}_{off}{p}_{3}- {\lambda }_{1}{p}_{1}-{k}_{on}{p}_{1}{p}_{2}$$

2 $${\frac{d{p}_{2}}{dt}=C}_{2}+{k}_{off}{p}_{3}- {\lambda }_{2}{p}_{2}-{k}_{on}{p}_{1}{p}_{2}$$

3 $${\frac{d{p}_{3}}{dt}={k}_{on}{p}_{1}{p}_{2}-k}_{off}{p}_{3}- {\lambda }_{3}{p}_{3}$$

In this system, ${p}_{1}$ monomer and ${p}_{2}$ monomer form a heterodimer ${p}_{3}$ with association and dissociation rate constants ${k}_{on}$ and ${k}_{off}$. The protein synthesis rates of ${p}_{1}$ and ${p}_{2}$ monomers are set as constants ${C}_{1}$ and ${C}_{2}$, respectively. Each protein specie ${p}_{i}$ has its degradation rate ${\lambda }_{i}$.

We assume that dimerization is much faster than synthesis and degradation based on the typical range of parameter values^42,57^, so equilibrium is reached instantaneously:

4 $${p}_{1}{p}_{2}{K}_{a}= {p}_{3}$$

where the association constant ${K}_{a}$ is the ratio of ${k}_{on}/{k}_{off}.$

To corroborate this assumption, we collected data about the rates of dimerization, protein synthesis and degradation from piror studies and justified the claim. The typical k_on_ of protein–protein complexes ranges from 10^5^ to 10^9^ M^-1^ s^-1^ (3.6 × 10^–1^–10^3^ nM^-1^ h^−1^)^57–59^ and the median protein concentration in cells is around ~ 300 nM as measured by a quantitative mass spectrometry study^42^. By using the median value (K_a_ = 0.05 nM^−1^) of binding affinity in PDBbind database^41^, we estimated the monomer concentration ~ 70 nM to give rise a total concentration 315 nM of a given protein. By multiplying the k_on_ by the square of monomer concentration, the typical dimerization rate ranges from 10^3^ to 10^7^ nM/h. Even if the smallest association rate constant is used for estimation, the slowest dimerization rate 1.746 × 10^3^ nM/h is still much faster than the median protein synthesis rate ~ 10^2^ nM/h in Table 1.

Let ${p}_{1}^{tot}$ and ${p}_{2}^{tot}$ be the total concentration of ${p}_{1}$ and ${p}_{2}$, respectively:

5 $${p}_{1}^{tot}= {{p}_{1}+p}_{3}$$

6 $${p}_{2}^{tot}= {{p}_{2}+p}_{3}$$

Thus, we can modify Eqs. (1) and (2) to:

7 $$\frac{d{{p}_{1}}^{tot}}{dt}={C}_{1}-{\lambda }_{1}{p}_{1}-{\lambda }_{3}{p}_{3}$$

8 $$\frac{d{{p}_{2}}^{tot}}{dt}={C}_{2}-{\lambda }_{2}{p}_{2}-{\lambda }_{3}{p}_{3}$$

The net rate of change for ${p}_{i}^{tot}$ is determined by the rate of inflow (monomer synthesis rate ${C}_{i}$) and rate of outflows (degradation rates when ${p}_{i}$ is in monomer form ${\lambda }_{i}{p}_{i}$ and in heterodimer ${\lambda }_{3}{p}_{3}$).

We solve these equations at steady-state where $\frac{d{{p}_{1}}^{tot}}{dt}=\frac{d{{p}_{2}}^{tot}}{dt}=0$.

First, we obtain ${p}_{2}$ in terms of ${p}_{1}$ and other parameters from (8) by replacing ${p}_{3}$ from (4):

$${C}_{2}-{\lambda }_{2}{p}_{2}-{\lambda }_{3}{p}_{1}{p}_{2}{K}_{a}=0$$

$${C}_{2}-{p}_{2}({\lambda }_{2}-{\lambda }_{3}{p}_{1}{K}_{a})=0$$

9 $${p}_{2}=\frac{{C}_{2}}{({\lambda }_{2}+{K}_{a}{\lambda }_{3}{p}_{1})}$$

Then, we solve ${p}_{1}$ from (7) by replacing ${p}_{3}$ and ${p}_{2}$ from (4) and (9):

$${C}_{1}-{\lambda }_{1}{p}_{1}-{\lambda }_{3}{p}_{1}(\frac{{C}_{2}}{({\lambda }_{2}+{K}_{a}{\lambda }_{3}{p}_{1})}){K}_{a}=0$$

Multiply both sides by $({\lambda }_{2}+{K}_{a}{\lambda }_{3}{p}_{1})$ so that:

$${C}_{1}{\lambda }_{2}-{\lambda }_{1}{\lambda }_{2}{p}_{1}-{K}_{a}{C}_{1}{\lambda }_{3}{p}_{1}-{K}_{a}{\lambda }_{1}{\lambda }_{3}{p}_{1}^{2}-{K}_{a}{C}_{2}{\lambda }_{3}{p}_{1}=0$$

Then, rearranging the equations:

$${K}_{a}{\lambda }_{1}{\lambda }_{3}{p}_{1}^{2}+{p}_{1}({\lambda }_{1}{\lambda }_{2}+{K}_{a}{C}_{1}{\lambda }_{3}+{K}_{a}{C}_{2}{\lambda }_{3})-{C}_{1}{\lambda }_{2}=0$$

Based on the quadratic formula, the solution for ${p}_{1}$ is $\frac{-\mathrm{b}\pm \sqrt{{\mathrm{b}}^{2}-4\mathrm{ac}}}{2\mathrm{a}}$, where $\mathrm{a}={K}_{a}{\lambda }_{1}{\lambda }_{3}$, $\mathrm{b}=({\lambda }_{1}{\lambda }_{2}+{K}_{a}{C}_{1}{\lambda }_{3}+{K}_{a}{C}_{2}{\lambda }_{3})$ and $\mathrm{c}=-{ \lambda }_{2}{C}_{1}$, so we obtained the solution to ${p}_{1}$ at steady-state:

$${p}_{1}=\frac{{K}_{a}{C}_{1}{\uplambda }_{3}-{K}_{a}{C}_{2}{\uplambda }_{3}-{\uplambda }_{1}{\uplambda }_{2} \pm \sqrt{{{K}_{a}}^{2}{{C}_{1}}^{2}{{\lambda }_{3}}^{2} + {{K}_{a}}^{2}{{c}_{2}}^{2}{{\uplambda }_{3}}^{2}-2 {{K}_{a}}^{2}{C}_{1}{C}_{2}{{\uplambda }_{3}}^{2}+2 {K}_{a} {C}_{1} {\uplambda }_{1}{\uplambda }_{2 }{\uplambda }_{3}+2 {K}_{a} {C}_{2} {\uplambda }_{1}{\uplambda }_{2 }{\uplambda }_{3}+ {{\uplambda }_{1}}^{2}{{\uplambda }_{2}}^{2} }}{2{\uplambda }_{1} {\uplambda }_{3} {K}_{a}}$$

A similar procedure gave a solution for ${p}_{2}$ at steady-state:

$${p}_{2}=\frac{-{K}_{a}{C}_{1}{\uplambda }_{3} + {K}_{a}{C}_{2}{\uplambda }_{3}-{\uplambda }_{1}{\uplambda }_{2} \pm \sqrt{{{K}_{a}}^{2}{{C}_{1}}^{2}{{\uplambda }_{3}}^{2} + {{K}_{a}}^{2}{{C}_{2}}^{2}{{\uplambda }_{3}}^{2}-2 {{K}_{a}}^{2}{C}_{1}{C}_{2}{{\uplambda }_{3}}^{2}+2 {K}_{a} {C}_{1} {\uplambda }_{1}{\uplambda }_{2 }{\uplambda }_{3}+2 {K}_{a} {C}_{2} {\uplambda }_{1}{\uplambda }_{2 }{\uplambda }_{3}+ {{\uplambda }_{1}}^{2}{{\uplambda }_{2}}^{2} }}{2{\uplambda }_{2} {\uplambda }_{3} {K}_{a}}$$

Given that the concentration of a protein cannot be negative, we selected $\frac{-\mathrm{b}+\sqrt{{\mathrm{b}}^{2}-4\mathrm{ac}}}{2\mathrm{a}}$ from both ${p}_{1}$ and ${p}_{2}$ solutions for further analysis.

Replacing the solutions to ${p}_{1}$ and ${p}_{2}$ in (4), we obtained a solution for ${p}_{3}$ at steady-state:

$${p}_{3}=\frac{{c}_{2}({K}_{a}{C}_{1}{\uplambda }_{3}-{K}_{a}{C}_{2}{\uplambda }_{3}-{\uplambda }_{1}{\uplambda }_{2} + \sqrt{{{K}_{a}}^{2}{{C}_{1}}^{2}{{\lambda }_{3}}^{2} + {{K}_{a}}^{2}{{c}_{2}}^{2}{{\uplambda }_{3}}^{2}-2 {{K}_{a}}^{2}{C}_{1}{C}_{2}{{\uplambda }_{3}}^{2}+2 {K}_{a} {C}_{1} {\uplambda }_{1}{\uplambda }_{2 }{\uplambda }_{3}+2 {K}_{a} {C}_{2} {\uplambda }_{1}{\uplambda }_{2 }{\uplambda }_{3}+ {{\uplambda }_{1}}^{2}{{\uplambda }_{2}}^{2} })}{{\uplambda }_{3}({K}_{a}{C}_{1}{\uplambda }_{3}- {K}_{a}{C}_{2}{\uplambda }_{3}+{\uplambda }_{1}{\uplambda }_{2}+ \sqrt{{{K}_{a}}^{2}{{C}_{1}}^{2}{{\uplambda }_{3}}^{2} + {{K}_{a}}^{2}{{C}_{2}}^{2}{{\uplambda }_{3}}^{2}-2 {{K}_{a}}^{2}{C}_{1}{C}_{2}{{\uplambda }_{3}}^{2}+2 {K}_{a} {C}_{1} {\uplambda }_{1}{\uplambda }_{2 }{\uplambda }_{3}+2 {K}_{a} {C}_{2} {\uplambda }_{1}{\uplambda }_{2 }{\uplambda }_{3}+ {{\uplambda }_{1}}^{2}{{\uplambda }_{2}}^{2} })}$$

### Heteromeric trimer

We denote ${p}_{1}^{tot}$, ${p}_{2}^{tot}$ and ${p}_{3}^{tot}$ as representing the total concentrations of ${p}_{1}$, ${p}_{2}$ and ${p}_{3}$, respectively. The three differential equations describing the system (Fig. 1B) based on our assumption that dimerization is faster than synthesis and degradation are:

10 $$\frac{d{{p}_{1}}^{tot}}{dt}={C}_{1}-{\lambda }_{1}{p}_{1}-{\lambda }_{12}{p}_{12}-{\lambda }_{123}{p}_{123}$$

11 $$\frac{d{{p}_{2}}^{tot}}{dt}={C}_{2}-{\lambda }_{2}{p}_{2}-{\lambda }_{12}{p}_{12}-{\lambda }_{123}{p}_{123}$$

12 $$\frac{d{{p}_{3}}^{tot}}{dt}={C}_{3}-{\lambda }_{3}{p}_{3}-{\lambda }_{123}{p}_{123}$$

13 $${{{p}_{1}p}_{2}K}_{1}={p}_{12}$$

14 $${{{p}_{12}p}_{3}K}_{2}={p}_{123}$$

where $${K}_{1}=\frac{{k}_{1on}}{{k}_{1off}}$$, $${K}_{2}=\frac{{k}_{2on}}{{k}_{2off}}$$.

At steady-state, Eqs. (10–12) can be rewritten by replacing.

${p}_{12}$ and ${p}_{123}$ from (13) and (14):

15 $${C}_{1}-{\lambda }_{1}{p}_{1}-{\lambda }_{12}{p}_{12}-{\lambda }_{12}{p}_{1}{p}_{2}{K}_{1}-{\lambda }_{123}{p}_{1}{p}_{2}{p}_{2}{K}_{1}{K}_{2}=0$$

16 $${C}_{2}-{\lambda }_{2}{p}_{2}-{\lambda }_{12}{p}_{12}-{\lambda }_{12}{p}_{1}{p}_{2}{K}_{1}-{\lambda }_{123}{p}_{1}{p}_{2}{p}_{2}{K}_{1}{K}_{2}=0$$

17 $${C}_{3}-{\lambda }_{3}{p}_{3}-{\lambda }_{123}{p}_{1}{p}_{2}{p}_{3}{K}_{1}{K}_{2}=0$$

This model cannot be analytically solved due to the non-linear term, so we numerically solved this nonlinear equation using parameter values within physiological ranges (Table 1).

### Open circuit without negative feedback control

To compare circuits with or without negative feedback regulation, we incorporated a transcription factor (${TF}_{i}$) and mRNA (${m}_{i}$) into our model:

18 $$\frac{d{m}_{t1}}{dt}={K}_{rt1}-{\lambda }_{mt1}{m}_{t1}$$

19 $$\frac{d{m}_{t2}}{dt}={K}_{rt2}-{\lambda }_{mt2}{m}_{t2}$$

20 $$\frac{d{TF}_{1}}{dt}={K}_{tt1}{m}_{t1}-{\lambda }_{tf1}{TF}_{1}$$

21 $$\frac{d{TF}_{2}}{dt}={K}_{tt2}{m}_{t2}-{\lambda }_{tf2}{TF}_{2}$$

where ${K}_{rti}$, ${K}_{tti}$, ${\lambda }_{mti}$ and ${\lambda }_{tfi}$ denote the transcription rate, translation rate constant, mRNA, and protein degradation rate constant of transcription factor $i$, respectively. Since we assume transcription and translation of the transcription factors are independent of the downstream mRNAs and proteins, we can treat their quantities as constants. At steady state ($\frac{d{m}_{ti}}{dt}=\frac{d{TF}_{i}}{dt}=0$), the concentration of ${m}_{ti}$ is $\frac{{K}_{rti}}{{\lambda }_{mti}}$ and ${TF}_{i}$ is $\frac{{K}_{tti}}{{\lambda }_{tfi}}{m}_{ti}$. By replacing ${m}_{ti}$ by $\frac{{K}_{rti}}{{\lambda }_{mti}}$, we can obtain the transcription factor concentration ${\mathrm{TF}}_{i}=\frac{{K}_{rti}{K}_{tti}}{{\lambda }_{mti}{\lambda }_{tfi}}$.

22 $$\frac{d{m}_{1}}{dt}={\mathrm{K}}_{r1}\frac{{TF}_{1}^{h}}{{K}_{tf1}^{h}+ {TF}_{1}^{h}}-{\uplambda }_{m1}{m}_{1}$$

23 $$\frac{d{m}_{2}}{dt}={\mathrm{K}}_{r2}\frac{{TF}_{2}^{h}}{{K}_{tf2}^{h}+ {TF}_{2}^{h}}-{\uplambda }_{m2}{m}_{2}$$

24 $$\frac{d{p}_{1}}{dt}={\mathrm{K}}_{t1}{m}_{1}+{k}_{off}{p}_{3}-{k}_{on}{p}_{1}{p}_{2}-{\lambda }_{1}{p}_{1}$$

25 $$\frac{d{p}_{2}}{dt}{=\mathrm{K}}_{t2}{m}_{2}+{k}_{off}{p}_{3}-{k}_{on}{p}_{1}{p}_{2}-{\lambda }_{2}{p}_{2}$$

26 $$\frac{d{p}_{3}}{dt}={k}_{on}{p}_{1}{p}_{2}-{k}_{off}{p}_{3}-{\lambda }_{3}{p}_{3}$$

where ${\mathrm{K}}_{ri}$, ${\mathrm{K}}_{ti}$, ${k}_{on}$, ${k}_{off}$, ${\mathrm{K}}_{tfi}$, and $h$ denote the transcription rate, translation rate constant, association rate constant, dissociation rate constant, TF-promoter binding affinity, and Hill coefficient, respectively. We solved the equations at steady-state so that the net reaction rates of all reactions equal zero. The system can be divided into two sub-systems, i.e., transcription factor synthesis and protein complex formation. By solving Eqs. (22) to (26), we obtained the levels of ${p}_{1}$ and ${p}_{2}$ mRNAs:

$${m}_{1}=\frac{{K}_{r1}{(TF1)}^{h}}{{\lambda }_{m1}({K}_{tf1}^{h}+{(TF1)}^{h})}$$

$${m}_{2}=\frac{{K}_{r2}{(TF2)}^{h}}{{\lambda }_{m2}({K}_{tf2}^{h}+{(TF2)}^{h})}$$

where $${\mathrm{TF}}_{1}=\frac{{K}_{rt1}{K}_{tt1}}{{\lambda }_{mt1}{\lambda }_{tf1}}$$ and $${\mathrm{TF}}_{2}=\frac{{K}_{rt2}{K}_{tt2}}{{\lambda }_{mt2}{\lambda }_{tf2}}$$.

Solving Eqs. (24) to (26) based on the quadratic formula, the solution for ${p}_{1}$ is $\frac{-\mathrm{b}\pm \sqrt{{\mathrm{b}}^{2}-4\mathrm{ac}}}{2\mathrm{a}}$, where $\mathrm{a}={k}_{on}{\lambda }_{1}{\lambda }_{3}$, $\mathrm{b}={k}_{on}{C}_{2}{\lambda }_{3}-{k}_{on}{C}_{1}{\lambda }_{3}+{\lambda }_{1}{\lambda }_{2}{k}_{off}$ and $\mathrm{c}=-{\lambda }_{2}{C}_{1}({\lambda }_{3}+{k}_{off})$, with ${C}_{i}$ being the translation rate of ${p}_{i}$, i.e., ${K}_{ti}{m}_{i}$.

The solutions to ${p}_{1}$ at steady-state, as well as ${p}_{2}$ and ${p}_{3}$, were derived with a similar fashion as described in the “Heterodimer” section above.

### Closed circuit with negative feedback control

Closed circuits exhibit feedback regulation exerted by monomers at the transcriptional level, so Eqs. (22) and (23) for the open circuit can be modified by adding the negative feedback term $\frac{K}{K+ {p}_{i}}$, where $K$ is the transcription factor and promoter association constant:

27 $$\frac{d{m}_{1}}{dt}={\mathrm{K}}_{r1}\frac{{TF}_{1}^{h}}{{K}_{tf1}^{h}+ {TF}_{1}^{h}}\frac{K}{K+ {p}_{1}}-{\uplambda }_{m1}{m}_{1}$$

28 $$\frac{d{m}_{2}}{dt}={\mathrm{K}}_{r2}\frac{{TF}_{2}^{h}}{{K}_{tf2}^{h}+ {TF}_{2}^{h}}\frac{K}{K+ {p}_{2}}-{\uplambda }_{m2}{m}_{2}$$

We solved the nonlinear equations numerically by assigning the parameters with values within physiological ranges (Table 1).

### Parameter estimation

All parameter values employed in our analysis are medians (where relevant) of their respective physiological ranges based on experimental data. Parameter data was obtained from PDBbind, (http://www.pdbbind.org.cn/)^41^ and all protein–protein interaction (PPI) dissociation constants were selected. The dissociation constant (${K}_{D}$) for a PPI is typically 1 ~ 1000 nM (permanent ~ transient)^60,61^, so we set a cutoff (${K}_{D}=1000 \mathrm{nM}$) to filter out weak PPIs considered transient interactions and thereby allowing us to focus on protein complexes. The physiological ranges for protein and mRNA half-lives were taken from two systematic studies on human cell lines^62,63^. Protein synthesis rates in human cell lines were estimated based on a quantitative study in which copy numbers of proteins per cell were quantified over time^42^. We converted the copy number to concentration based on a typical cell volume of plasma cells (~ 2000 μm^3^). All data employed are plotted in Fig. S2. The transcription and translation rates were adjusted so that the final protein synthesis rate fell within their respective physiological ranges. Values for the repressor-promoter dissociation constant and cooperativity of promoter activity in vivo were chosen based on previous studies^64,65^.

### Tolerance range

We propose a tolerance range to measure the ranges of the monomer synthesis rates maintaining subunit stoichiometry. Specifically, we fixed the ${p}_{2}$ synthesis rate and then varied the ${p}_{1}$ synthesis rate, which established upper and lower limits of the ${p}_{1}$ synthesis rate when dimer ${p}_{3}$ abundance exceeds that of monomers ${p}_{1}$ and ${p}_{2}$. The tolerance range is defined as:

$$Tolerance range=\left\{\begin{array}{l}0, Upper limit\le Lower limit\\ {\mathrm{log}}_{2}\frac{Upper limit}{Lower limit}, Upper limit>Lower limit\end{array}\right.$$

### Tolerance score

In line with tolerance range, we propose a tolerance score to measure the robustness of stoichiometry maintenance by quantifying the difference between dimer and monomer concentrations within the physiological range of monomer synthesis variations ($\le$twofold). We also fixed the ${p}_{2}$ synthesis rate (${C}_{2}$) and then varied the ${p}_{1}$ synthesis rate (${C}_{1}$) from 0 to twofold of ${C}_{2}$. The tolerance score is defined as:

$$Tolerance score={\int }_{0}^{2{C}_{2}}{(p}_{3}-\mathit{max}\left({p}_{1}, {p}_{2}\right))d{C}_{1}$$

The value of integral from ${C}_{1}=0$ to ${C}_{1}$=$2{C}_{2}$ of dimer minus the more abundant monomer can be negative if dimer is less than the monomer.

### Selection of eight characteristics for a protein complex formation system

We assessed the simulated data generated from our four circuits (Fig. 3A–D) according to eight systems characteristics. There are two scenarios for the five steady-state properties: stoichiometrically balanced (${p}_{1}$ and ${p}_{2}$ synthesis rates are identical) and stoichiometrically imbalanced (${p}_{1}$ synthesis rate is 0.5-fold or twofold the ${p}_{2}$ synthesis rate). The fold-change in synthesis rate was achieved by assigning the indicated fold-change to the transcription rate constant (${K}_{r}$). We obtained solutions for ${m}_{1}$, ${m}_{2}$, ${p}_{1}$, ${p}_{2}$, and ${p}_{3}$ in the two scenarios. When the ${p}_{1}$ and ${p}_{2}$ synthesis rates are identical, (1) “Efficiency” is obtained by dividing ${p}_{3}$ by ${m}_{1}$, and (2) “Dimer ratio 1” is the ratio of ${p}_{3}$ to the total protein concentration (${p}_{1}+{p}_{2}+{p}_{3}$). (3) “Dimer ratio 2” is the ratio of ${p}_{3}$ to (${p}_{1}+{p}_{2}+{p}_{3}$) when the ${p}_{1}$ synthesis rate is twofold that of ${p}_{2}$. (4) “Upward controllability” is obtained by calculating the fold-change of ${p}_{3}$ relative to the twofold increase in the ${p}_{1}$ synthesis rate, and (5) “Downward controllability” is obtained by calculating the fold-change of ${p}_{3}$ relative to the 0.5-fold decrease in the ${p}_{1}$ synthesis rate. For the three kinetic properties, we used ODE solver in SciPy, a scientific library for Python, to solve the differential equations with the indicated parameter values, the initial state of each reactant, a duration time of 1,200,000 s, and a time interval of 0.5 s. Accordingly, (6) “Upward response time” is the duration for dimer quantities to reach 200 from 0 nM based on an abrupt increase in transcription rates and an initial state that all reactants are 0 nM. Similarly, (7) “Downward response time” is the duration for dimer quantities to fall below 50% of their steady-state levels, with an abrupt termination of translation rates and an initial state that all reactants are at steady-state levels. Finally, (8) “Recovery time” is the duration for ${p}_{1}$ monomer to return to its steady-state value after an impulsive perturbation (fivefold its steady-state value), with all other reactants starting from their steady-state values.

### Comparing system characteristics over parameter configurations

The characteristic values of a circuit depend on its parameter configurations. To compare the performance of each pair of circuits, we generated simulated data with combinations of parameter values within physiologically relevant ranges (Table 1) and then counted the proportion of parameter configurations in which one circuit was superior to the other. We considered four parameters in the models: (1) synthesis rate ($C$); (2) protein–protein interaction equilibrium constant (${K}_{a}$); (3) cooperative stability ($\lambda$); and (4) negative feedback strength ($K$). Next, we chose 10 values evenly distributed across the physiologically relevant range for each parameter. The four circuits have different numbers of parameters and parameter configurations: 10^2^ for Circuit 1 ($C$ and ${K}_{a}$), 10^3^ for Circuits 2 and 3 ($C$, ${K}_{a}$, $\lambda$ or $K$), and 10^4^ for Circuit 4 ($C$, ${K}_{a}$, $\lambda$, $K$). To standardize the numbers of parameter configurations for circuit comparison, we generated degenerate configurations ($\lambda =0$ or $K=0$) 10 or 100 times so as to produce 10^4^ comparison outcomes for each pair of circuits.

### Performance score

We assigned each protein complex circuit a performance score for each system characteristic. A performance score is typically deduced from simultaneous comparison of all circuits. However, since each circuit has a different number of parameters, simultaneous comparison of all our circuits may not be appropriate. Instead, we converted the pairwise comparison results into a performance score by first evaluating the probability ${P}_{i>j}$ for a circuit pair $(i,j)$ as the fraction of the 10^4^ parameter configurations where Circuit $i$ is superior to Circuit $j$. Then, we calculated the probabilities of all $4!=24$ possible rankings of the four circuits by combining the pairwise probabilities compatible with the ranking order:

29 $$P({i}_{1},{i}_{2},{i}_{3},{i}_{4})\propto \prod_{\begin{array}{c}{j}_{1},{j}_{2}\in \left\{{i}_{1},{i}_{2},{i}_{3},{i}_{4}\right\}\\ {j}_{1}\prec {j}_{2} \end{array}}{ P}_{ {j}_{1}> {j}_{2}}$$

where ${j}_{1}\prec {j}_{2}$ denotes that ${j}_{1}$ precedes ${j}_{2}$ in the ranking order. Next, based on the ranking probabilities, we obtained the probability mass function (PMF) ${P}_{i}(r=j)$ to denote the probability that Circuit $i$ has rank $j$, representing the sum of all compatible rank probabilities:

30 $${P}_{i}\left(r=j\right)=\sum_{\left({i}_{1},{i}_{2},{i}_{3},{i}_{4}\right):{i}_{j}=i}P({i}_{1},{i}_{2},{i}_{3},{i}_{4})$$

Finally, we evaluated the cumulative mass function (CMF) ${P}_{i}(r\le j)$ and used the area under CMF as the performance score.

### Consent for publication

All authors have read and approved the manuscript.

## Supplementary Information

Supplementary Information 1.

Supplementary Information 2.

Supplementary Information 3.

Supplementary Information 4.

Supplementary Information 5.

Supplementary Information 6.

Supplementary Information 7.

Supplementary Information 8.

Supplementary Information 9.

Supplementary Information 10.

## Data Availability

The work is derived from simulated data. The source codes of building quantitative models and running simulations are provided in Supplementary File S2.

## References

[1] Alberts B (1998). The cell as a collection of protein machines: preparing the next generation of molecular biologists. Cell.

[2] Burke D, Gasdaska P, Hartwell L (1989). Dominant effects of tubulin overexpression in Saccharomyces cerevisiae. Mol. Cell. Biol..

[3] Papp B, Pál C, Hurst LD (2003). Dosage sensitivity and the evolution of gene families in yeast. Nature.

[4] Abruzzi KC (2002). Protection from free β-tubulin by the β-tubulin binding protein Rbl2p. Mol. Cell. Biol..

[5] Harper JW, Bennett EJ (2016). Proteome complexity and the forces that drive proteome imbalance. Nature.

[6] Dandekar T (1998). Conservation of gene order: a fingerprint of proteins that physically interact. Trends Biochem. Sci..

[7] Wells JN, Bergendahl LT, Marsh JA (2016). Operon gene order is optimized for ordered protein complex assembly. Cell Rep..

[8] Lee JW, Zemojtel T, Shakhnovich E (2009). Systems-level evidence of transcriptional co-regulation of yeast protein complexes. J. Comput. Biol..

[9] Webb EC, Westhead DR (2009). The transcriptional regulation of protein complexes; a cross-species perspective. Genomics.

[10] Li G-W (2014). Quantifying absolute protein synthesis rates reveals principles underlying allocation of cellular resources. Cell.

[11] Taggart JC, Li G-W (2018). Production of protein-complex components is stoichiometric and lacks general feedback regulation in eukaryotes. Cell Syst..

[12] Jaenisch R, Bird A (2003). Epigenetic regulation of gene expression: how the genome integrates intrinsic and environmental signals. Nat. Genet..

[13] Wang Y (2015). Mechanism of alternative splicing and its regulation. Biomed. Rep..

[14] Yeh CW (2021). The C-degron pathway eliminates mislocalized proteins and products of deubiquitinating enzymes. EMBO J..

[15] Holt LJ (2012). Regulatory modules: coupling protein stability to phopshoregulation during cell division. FEBS Lett..

[16] Zhou Z (2016). Codon usage is an important determinant of gene expression levels largely through its effects on transcription. Proc. Natl. Acad. Sci..

[17] Heck AM, Wilusz J (2018). The interplay between the RNA decay and translation machinery in eukaryotes. Cold Spring Harbor Perspect. Biol..

[18] Sharma D (2021). The kinetic landscape of an RNA-binding protein in cells. Nature.

[19] Kamp HD, Higgins DE (2011). A protein thermometer controls temperature-dependent transcription of flagellar motility genes in *Listeria monocytogenes*. PLoS Pathog..

[20] Fuller S, Gaitanaki C, Sugden P (1989). Effects of increasing extracellular pH on protein synthesis and protein degradation in the perfused working rat heart. Biochem. J..

[21] Habibi I, Emamian ES, Abdi A (2014). Quantitative analysis of intracellular communication and signaling errors in signaling networks. BMC Syst. Biol..

[22] Gonçalves E (2017). Widespread post-transcriptional attenuation of genomic copy-number variation in cancer. Cell Syst..

[23] Tayebi N (2003). Reciprocal and nonreciprocal recombination at the glucocerebrosidase gene region: implications for complexity in Gaucher disease. Am. J. Hum. Genet..

[24] Scazzari M, Amm I, Wolf DH (2015). Quality control of a cytoplasmic protein complex: chaperone motors and the ubiquitin-proteasome system govern the fate of orphan fatty acid synthase subunit Fas2 of yeast. J. Biol. Chem..

[25] Natarajan N (2020). Quality control of protein complex assembly by a transmembrane recognition factor. Mol. Cell.

[26] Mueller S (2015). Protein degradation corrects for imbalanced subunit stoichiometry in OST complex assembly. Mol. Biol. Cell.

[27] Lam YW (2007). Analysis of nucleolar protein dynamics reveals the nuclear degradation of ribosomal proteins. Curr. Biol..

[28] Sung M-K (2016). A conserved quality-control pathway that mediates degradation of unassembled ribosomal proteins. Elife.

[29] Ishikawa K (2017). Post-translational dosage compensation buffers genetic perturbations to stoichiometry of protein complexes. PLoS Genet..

[30] McShane E (2016). Kinetic analysis of protein stability reveals age-dependent degradation. Cell.

[31] Buchler NE, Gerland U, Hwa T (2005). Nonlinear protein degradation and the function of genetic circuits. Proc. Natl. Acad. Sci..

[32] Zhang F (2010). Effects of nonlinear degradation of protein on the oscillatory dynamics in a simple gene regulatory network. Phys. A Stat. Mech. Appl..

[33] Peng Y (2015). Temperature compensation via cooperative stability in protein degradation. Phys. A Stat. Mech. Appl..

[34] Veitia RA (2003). Nonlinear effects in macromolecular assembly and dosage sensitivity. J. Theor. Biol..

[35] Schuetz R (2012). Multidimensional optimality of microbial metabolism. Science.

[36] Lyttle DN (2017). Robustness, flexibility, and sensitivity in a multifunctional motor control model. Biol. Cybern..

[37] Mahmoud MS (2018). Advanced control design with application to electromechanical systems.

[38] Wang L (2009). Bistable switches control memory and plasticity in cellular differentiation. Proc. Natl. Acad. Sci..

[39] Bell-Pedersen D (2005). Circadian rhythms from multiple oscillators: lessons from diverse organisms. Nat. Rev. Genet..

[40] Koyama M, Pujala A (2018). Mutual inhibition of lateral inhibition: a network motif for an elementary computation in the brain. Curr. Opin. Neurobiol..

[41] Liu Z (2015). PDB-wide collection of binding data: current status of the PDBbind database. Bioinformatics.

[42] Liu T-Y (2017). Time-resolved proteomics extends ribosome profiling-based measurements of protein synthesis dynamics. Cell Syst..

[43] Brophy JA, Voigt CA (2014). Principles of genetic circuit design. Nat. Methods.

[44] Verbič A, Praznik A, Jerala R (2020). A guide to the design of synthetic gene networks in mammalian cells. FEBS J..

[45] Munteanu A (2014). Design principles of stripe-forming motifs: the role of positive feedback. Sci. Rep..

[46] Chau AH (2012). Designing synthetic regulatory networks capable of self-organizing cell polarization. Cell.

[47] Romero F (2018). Lipid synthesis is required to resolve endoplasmic reticulum stress and limit fibrotic responses in the lung. Am. J. Resp. Cell Mol. Biol..

[48] Choy MS (2015). Structural and functional analysis of the GADD34: PP1 eIF2α phosphatase. Cell Rep..

[49] Del Vecchio D, Dy AJ, Qian Y (2016). Control theory meets synthetic biology. J. R. Soc. Interf..

[50] Cardarelli L, Maxwell KL, Davidson AR (2011). Assembly mechanism is the key determinant of the dosage sensitivity of a phage structural protein. Proc. Natl. Acad. Sci..

[51] Cerychova R, Pavlinkova G (2018). HIF-1, metabolism, and diabetes in the embryonic and adult heart. Front. Endocrinol..

[52] Bertolini M (2021). Interactions between nascent proteins translated by adjacent ribosomes drive homomer assembly. Science.

[53] Shemorry A, Hwang C-S, Varshavsky A (2013). Control of protein quality and stoichiometries by N-terminal acetylation and the N-end rule pathway. Mol. Cell.

[54] Varshavsky A (2019). N-degron and C-degron pathways of protein degradation. Proc. Natl. Acad. Sci..

[55] Lin H-C (2018). C-terminal end-directed protein elimination by CRL2 ubiquitin ligases. Mol. Cell.

[56] Trauth J (2019). Synthetic control of protein degradation during cell proliferation and developmental processes. ACS Omega.

[57] Schreiber G, Haran G, Zhou H-X (2009). Fundamental aspects of protein–protein association kinetics. Chem. Rev..

[58] Keeble AH (2008). Experimental and computational analyses of the energetic basis for dual recognition of immunity proteins by colicin endonucleases. J. Mol. Biol..

[59] Qin S, Pang X, Zhou H-X (2011). Automated prediction of protein association rate constants. Structure.

[60] Perkins JR (2010). Transient protein-protein interactions: structural, functional, and network properties. Structure.

[61] Xing S (2016). Techniques for the analysis of protein-protein interactions in vivo. Plant Physiol..

[62] Boisvert F-M (2012). A quantitative spatial proteomics analysis of proteome turnover in human cells. Mol. Cell. Proteom..

[63] Narula A (2019). Coding regions affect mRNA stability in human cells. RNA.

[64] Gerland U, Moroz JD, Hwa T (2002). Physical constraints and functional characteristics of transcription factor–DNA interaction. Proc. Natl. Acad. Sci..

[65] Wolf DM, Eeckman FH (1998). On the relationship between genomic regulatory element organization and gene regulatory dynamics. J. Theor. Biol..

