# Supplementary figures and images for: Cooperative stability renders protein complex formation more robust and controllable
Source: Sci Rep. 2022 Jun 21;12:10490. doi: 10.1038/s41598-022-14362-z (PMC9213465; doi:10.1038/s41598-022-14362-z)

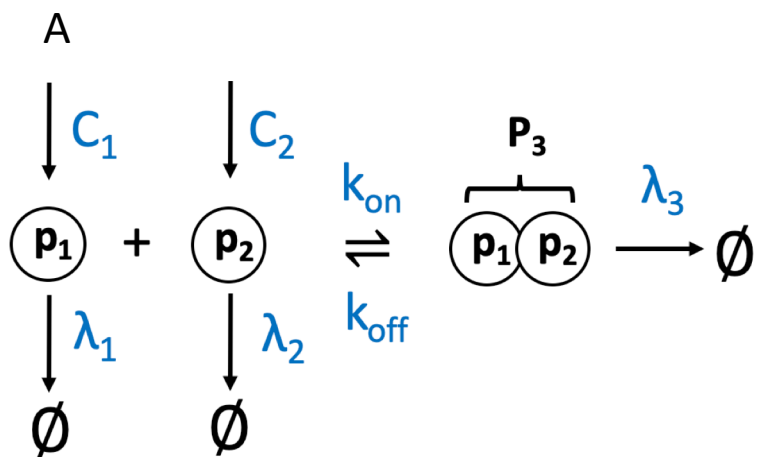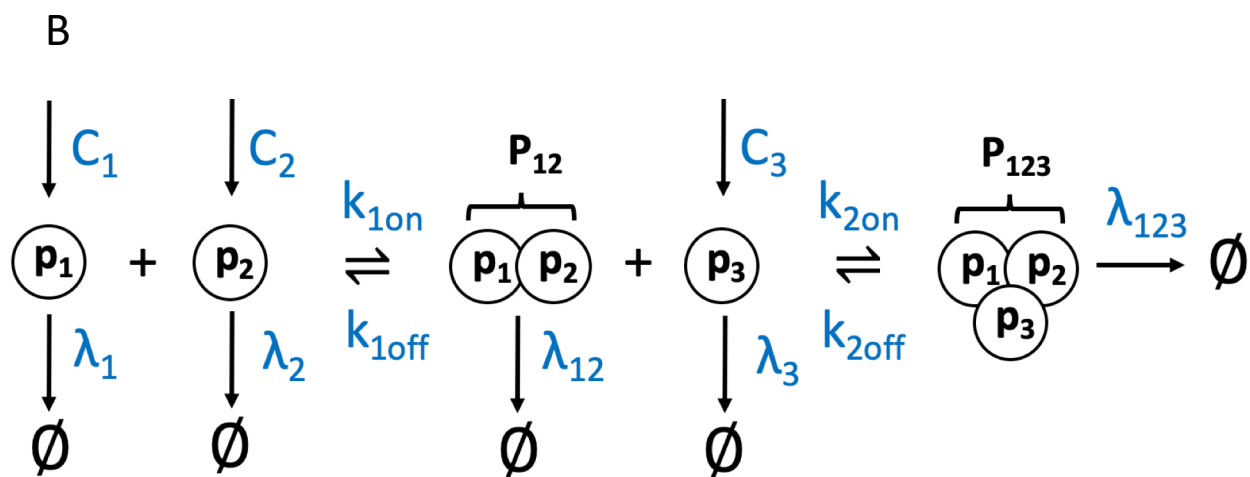

Supplement: Supplementary file 1 — Supplementary Information 1. [file 41598_2022_14362_MOESM1_ESM.pdf]

A

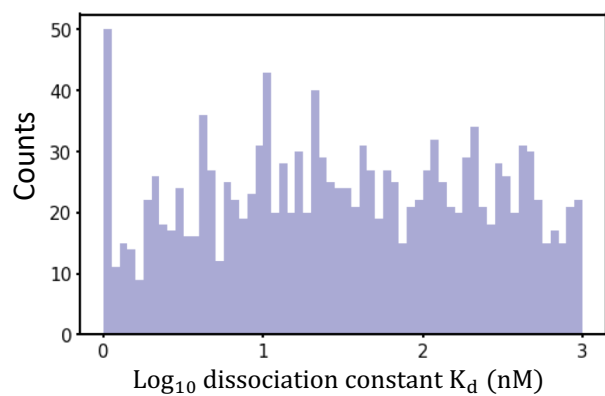

B

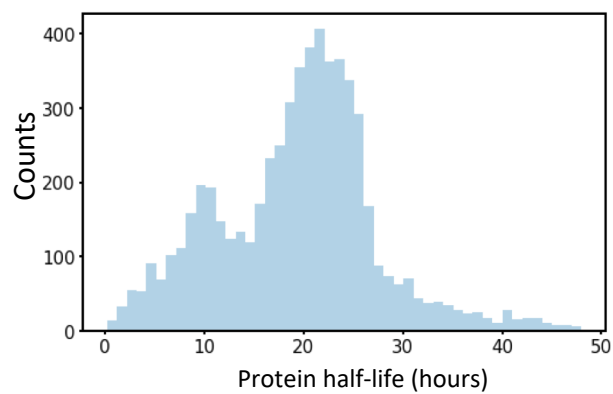

C

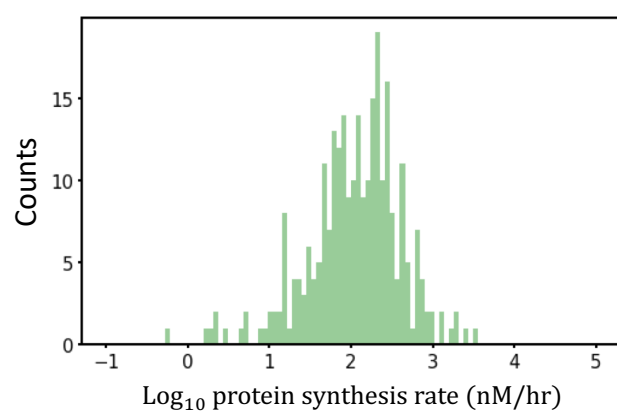

D

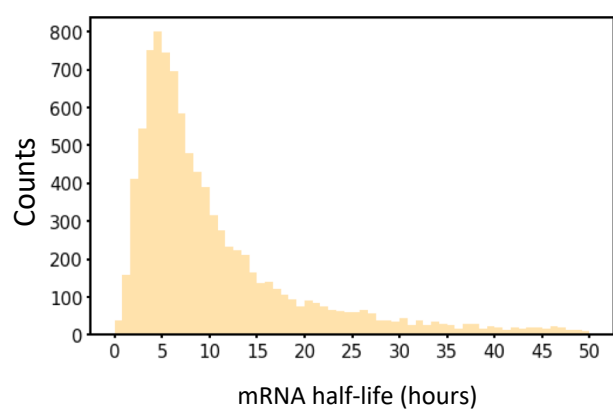

Supplement: Supplementary file 2 — Supplementary Information 2. [file 41598_2022_14362_MOESM2_ESM.pdf]

A

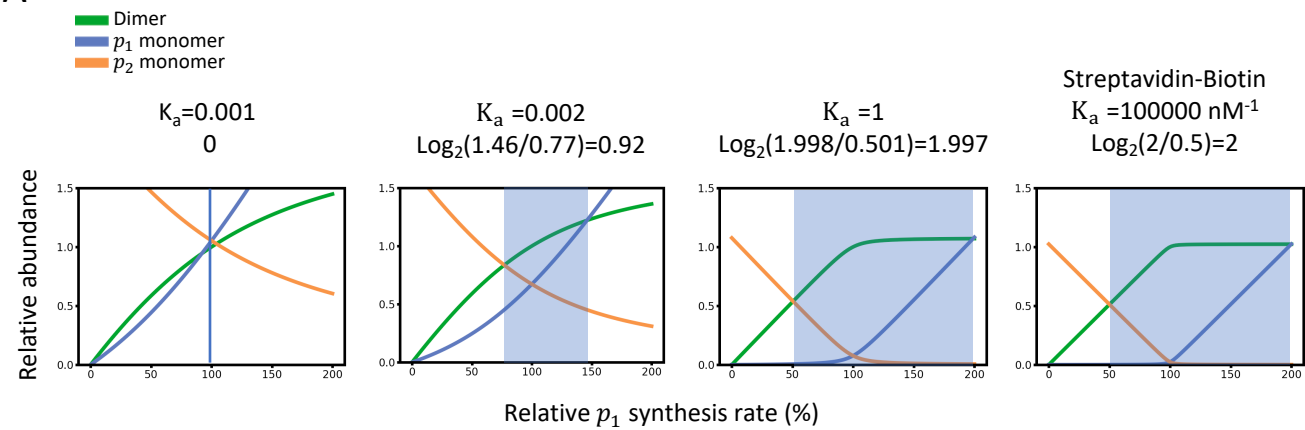

B

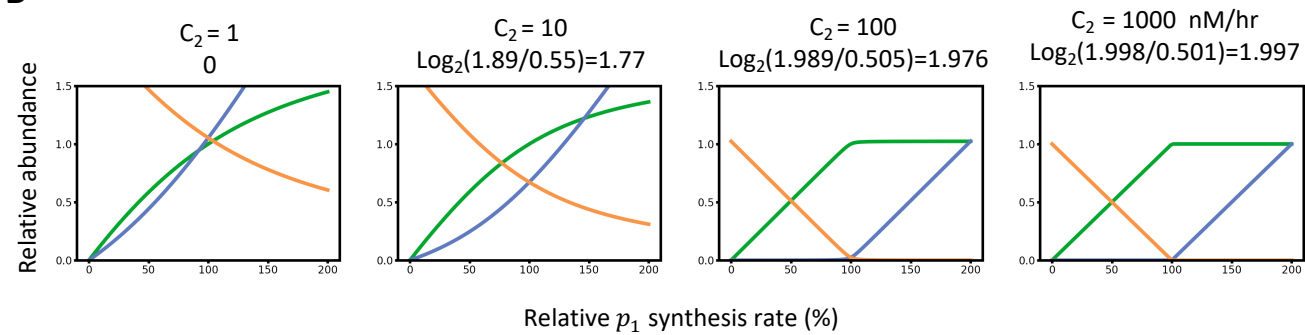

Supplement: Supplementary file 3 — Supplementary Information 3. [file 41598_2022_14362_MOESM3_ESM.pdf]

■ Dimer  
■  $p_1$  monomer  
■  $p_2$  monomer

**A**

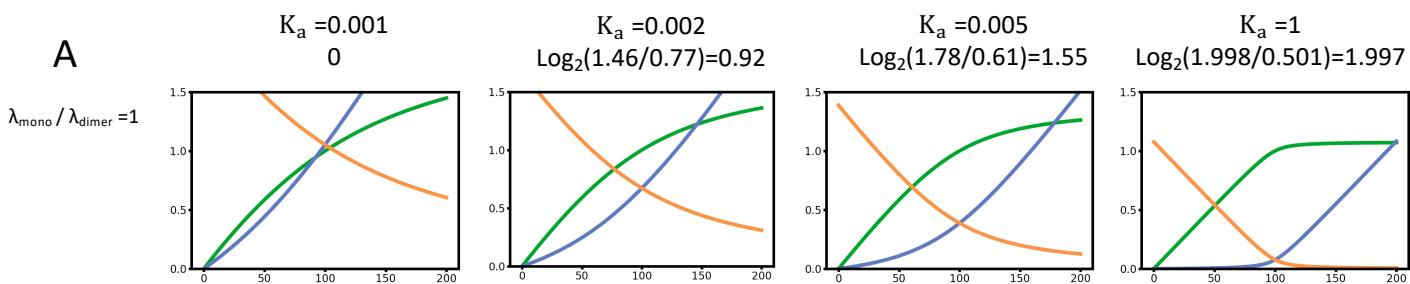

**B**

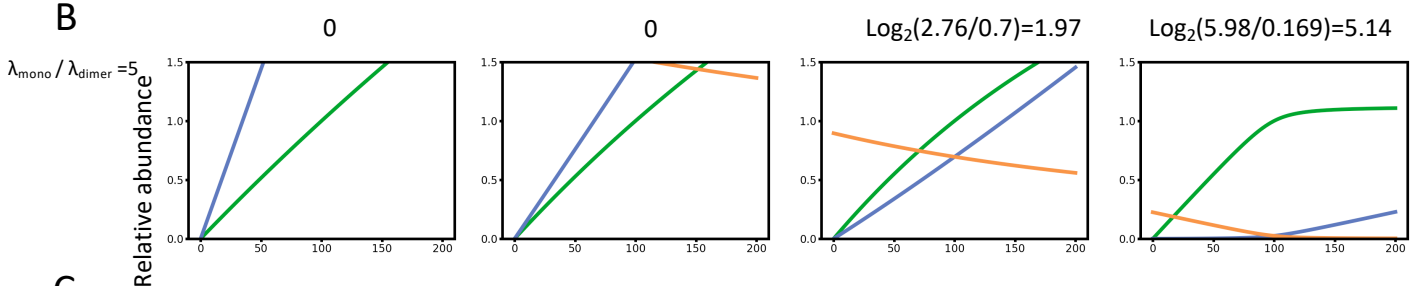

**C**

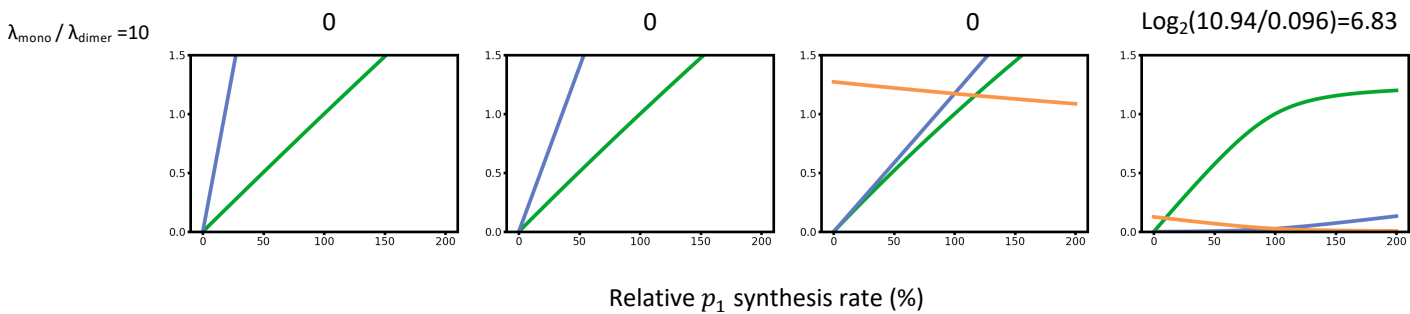

Supplement: Supplementary file 4 — Supplementary Information 4. [file 41598_2022_14362_MOESM4_ESM.pdf]

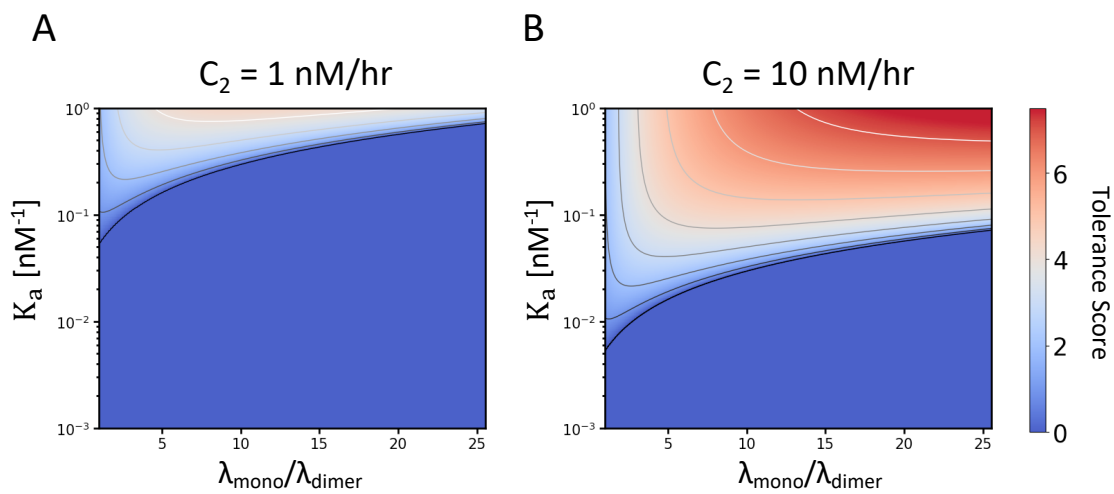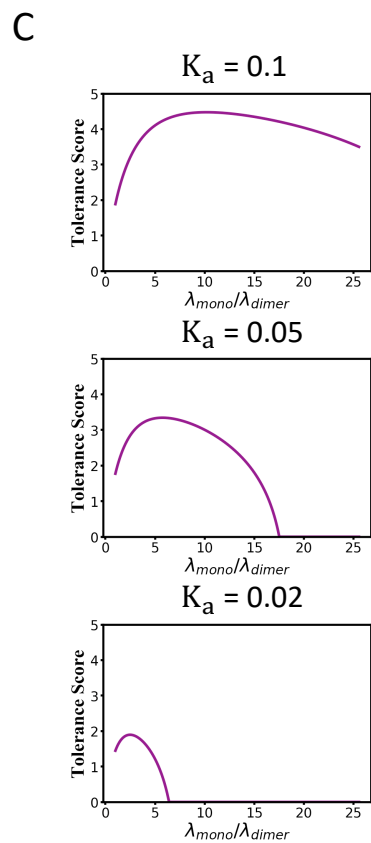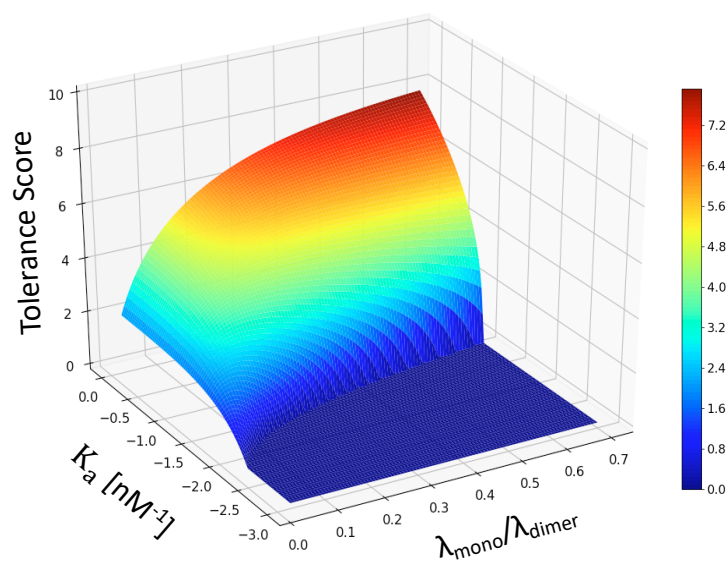

Supplement: Supplementary file 5 — Supplementary Information 5. [file 41598_2022_14362_MOESM5_ESM.pdf]

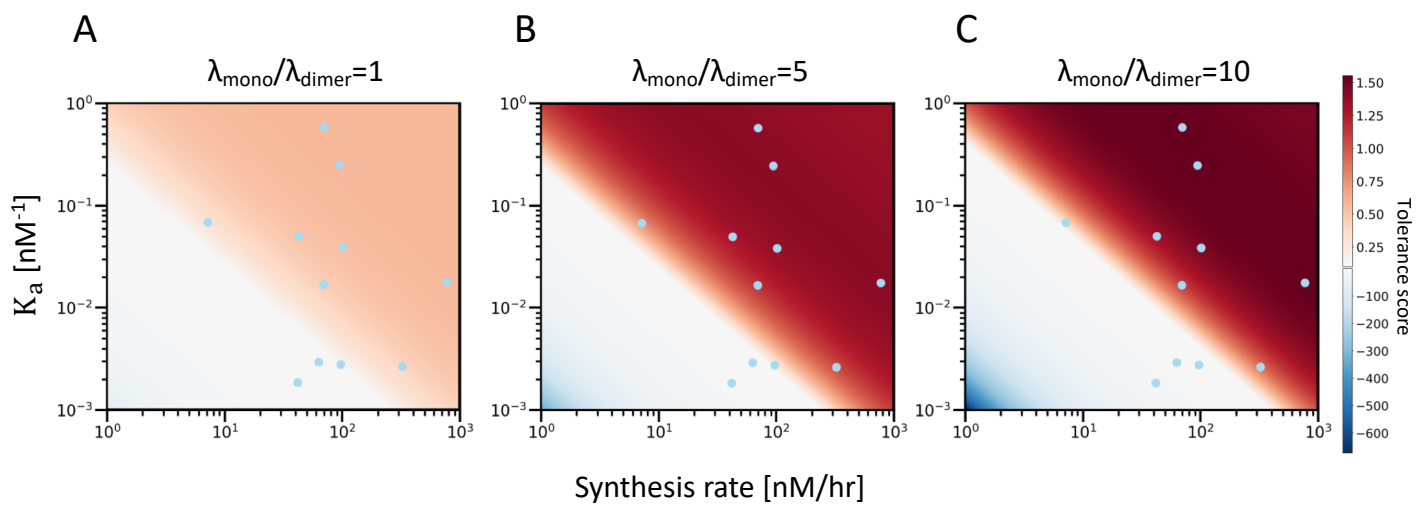

Supplement: Supplementary file 6 — Supplementary Information 6. [file 41598_2022_14362_MOESM6_ESM.pdf]

A

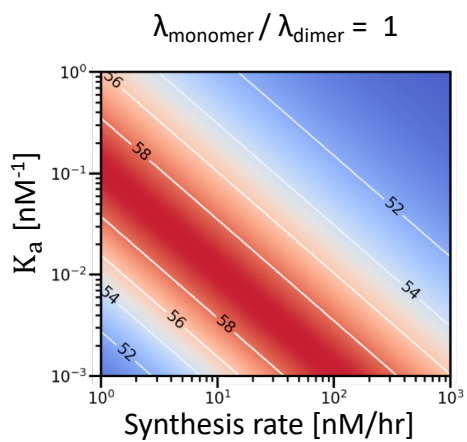

B

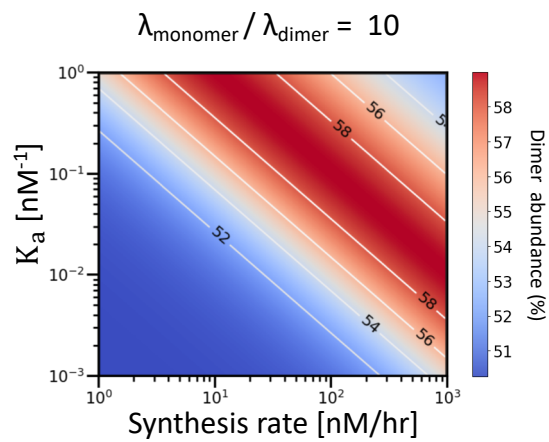

C

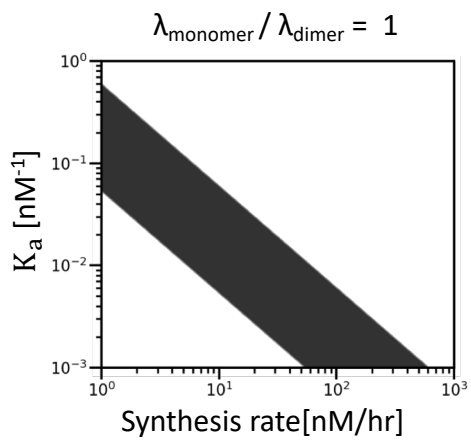

D

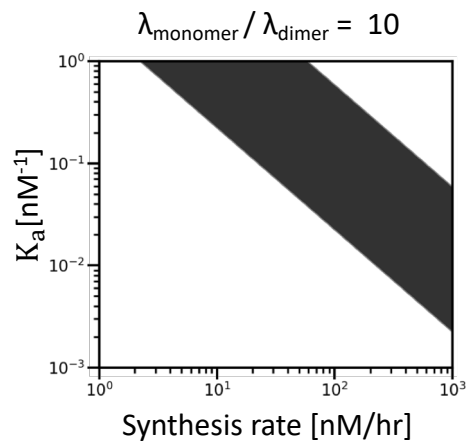

Supplement: Supplementary file 8 — Supplementary Information 8. [file 41598_2022_14362_MOESM8_ESM.pdf]
