# Supplementary material for: Cooperative stability renders protein complex formation more robust and controllable
Source: Sci Rep. 2022 Jun 21;12:10490. doi: 10.1038/s41598-022-14362-z (PMC9213465; doi:10.1038/s41598-022-14362-z)
Supplement: Supplementary file 7 — Supplementary Information 7. [file 41598_2022_14362_MOESM7_ESM.pdf]

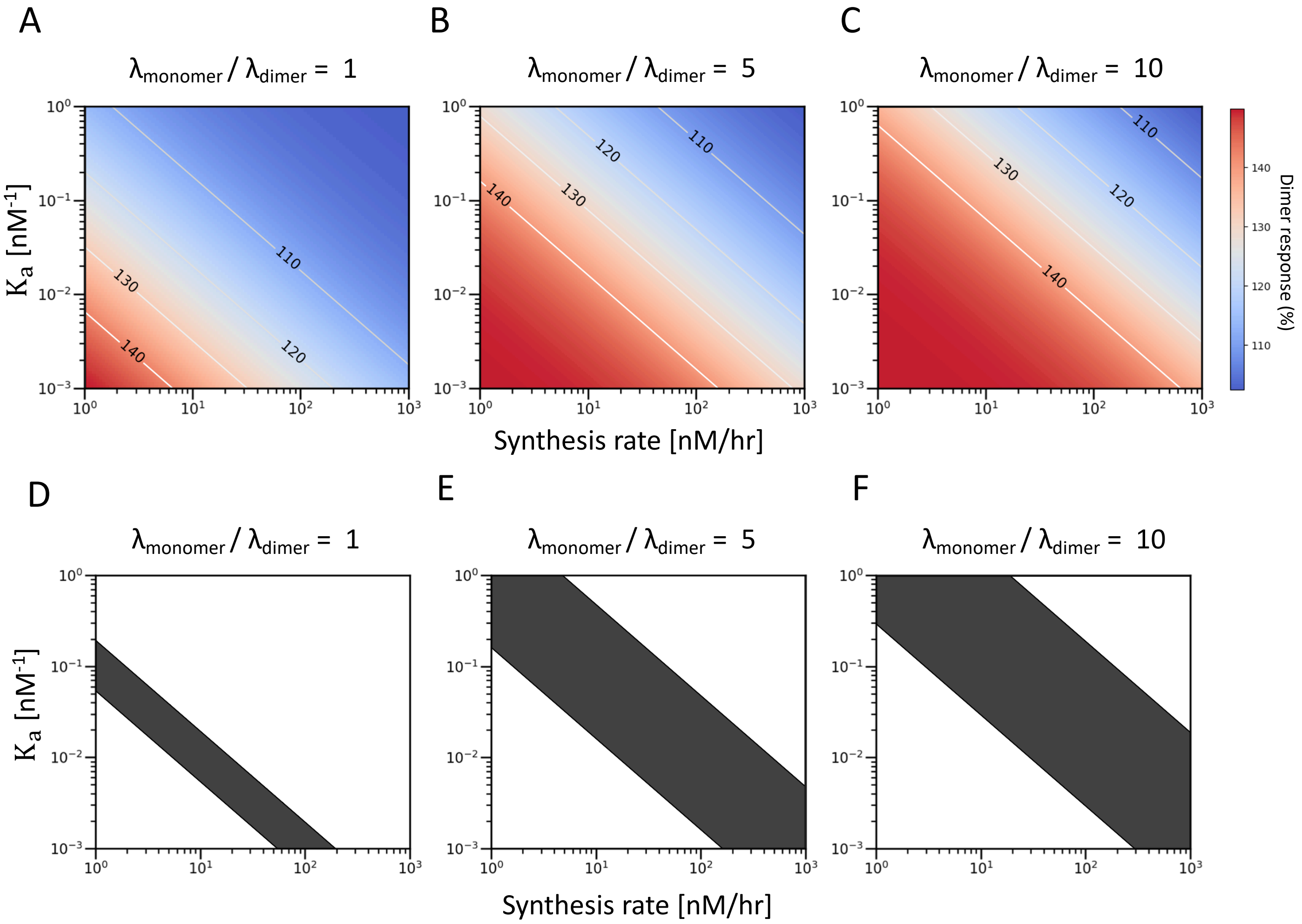
A
B
C
D
E
F
λmonomer / λdimer = 1
λmonomer / λdimer = 5
λmonomer / λdimer = 10
Ka [nM-1]
Synthesis rate [nM/hr]
Dimer response (%)
110
120
130
140
