# Supplementary material for: Cooperative stability renders protein complex formation more robust and controllable
Source: Sci Rep. 2022 Jun 21;12:10490. doi: 10.1038/s41598-022-14362-z (PMC9213465; doi:10.1038/s41598-022-14362-z)
Supplement: Supplementary file 9 — Supplementary Information 9. [file 41598_2022_14362_MOESM9_ESM.pdf]

Cooperative stability renders protein complex formation more robust and controllable

Kuan-Lun Hsu[1], Hsueh-Chi S Yen[1], Chen-Hsiang Yeang[2*]

1. Institute of Molecular Biology, Academia Sinica, 128 Academia Road, Section 2, Taipei, Taiwan
2. Institute of Statistical Science, Academia Sinica, 128 Academia Road, Section 2, Taipei, Taiwan

*: corresponding author: chyeang@stat.sinica.edu.tw

Supplementary Figure and File legends

Fig. S1. Schematics of heteromeric protein complex formation models.

(A) For heterodimers, the two distinct monomers p_1 and p_2 are synthesized at constant synthesis rates C_1 and C_2, respectively. These two subunits associate together to form a heterodimer p_3 and this reaction is reversible according to association/dissociation rate constants k_{on} and k_{off}. Each species (p_1 , p_2 , p_3) is degraded with degradation rate constants λ_1 , λ_2 ,and λ_3, respectively. (B) For heteromeric trimers, three distinct monomers p_1 , p_2 and p_3 are synthesized at constant rates C_1 , C_2 and C_3, respectively. Trimer formation is a two-step sequential and reversible heterodimerization process, whereby p_1 and p_2 first form the heterodimer p_{12}, and then associated with p_3 to form the p_{123} trimer, with association/dissociation rate constants k_{ion} and $k_{ioff}$and i being the order of the steps. Each protein species (p_1 , p_2 , p_3, p_{12} and p_{123}) is degraded according to degradation rate constants λ_1 , λ_2 , λ_3 , λ_{12} and λ_{123}, respectively.

Fig. S2. Distributions and physiological ranges for each model parameter.

(A) The distribution of dissociation constant K_d for protein-protein interactions downloaded from the PDBbind database [41]. The x-axis is in logarithmic scale. (B) The distribution of protein half-lives (in hours) in human cells, as measured by high-throughput SILAC analysis in a prior study [62]. (C) The distribution of protein synthesis rates in a human cell line, as measured by high-throughput SILAC analysis and Intensity Based Absolute Quantification (iBAQ), to determine the protein copy number produced during a given time period [42]. The concentration is inferred from an average cell volume of 2000 μm^3. (D) The distribution of mRNA half-lives (in hours) in human cells, as measured by 4-U labeling pulse-chase experiments in a previous study [63].

Fig. S3. Systematic constraints on a protein complex formation system lacking cooperative stability.

Quantification of the range of synthesis rate variation for dimers > monomers with different binding affinities (K_a) and a fixed p_2 synthesis rate. We defined this range (shaded area in A) as the tolerance score (see Materials and Methods). Response curves of dimer (green), p_1 (blue) and p_2 (orange) are plotted as described in Fig. 2A-C with different values for K_a (A) and p_2 synthesis rates (B). In (A), only K_a varies as indicated, while the other parameters are fixed (C_2 = 50 nM/hr, C_1 = 0~2x C_2, $\lambda_{momomer}/\lambda_{dimer} = 1$). In (B), only C_2 varies as indicated, while the other parameters are fixed (K_a = 0.05 nM^{-1}, C_1 = 0~2x C_2, $\lambda_{momomer}/\lambda_{dimer} = 1$). Respective tolerance ranges are shown above the figures.

Fig. S4. The effectiveness of cooperative stability in terms of maintaining the stoichiometric balance of complex subunits depends on the association constant.

Quantification of the range of synthesis rate variation for dimer is greater than for monomer with combinations of different K_a and $\lambda_{momomer}/\lambda_{dimer}$. (A-C) Response curves of dimer (green), p_1 (blue) and p_2 (orange) are plotted as described in Fig. 2A-C, and respective tolerance ranges are shown above the figures. K_a varies from 0.001 to 1 nM^{-1} as indicated. Different ratios of monomer and dimer degradation rates (1, 5 and 10) are presented.

Fig. S5. The tolerance range depends on $\lambda_{momomer}/\lambda_{dimer}$, K_a and C_2 in a non-monotonic fashion.

The tolerance range for different combinations of C_2 synthesis rate, association constant K_a and $\lambda_{\mathrm{momomer}}/\lambda_{\mathrm{dimer}}$. Heatmaps of tolerance ranges within K_a and $\lambda_{\mathrm{momomer}}/\lambda_{\mathrm{dimer}}$ physiological ranges are plotted with C_2 = 1 nM/hr (A) and C_2 = 10 nM/hr (B), respectively. (C) Dependency of the tolerance range on $\lambda_{\mathrm{momomer}}/\lambda_{\mathrm{dimer}}$ and K_a is visualized in a 3D surface (right) and with 2D sections (left) for three K_a values. Note, the C_2 synthesis rate is fixed at 10 nM/hr.

Fig. S6. The distribution of association constants and protein synthesis rates of protein complexes

(A-C) The experimentally measured association constants and protein synthesis rates of 11 protein complexes are collected from PDBbind[41] and a quantitative proteomic study[42]. The distribution of these protein complexes in parameter space was plotted along with the tolerance score heatmaps as Fig. 1G-I. Each sky-blue dot represented the parameter configuration of one protein complex.

Fig. S7. Cooperative stability enhances the robustness and upward controllability of protein complex formation systems.

(A-C) Heatmaps of dimer abundance responses to a 150% synthesis rate increase within the physiological range. The x-axes represent the fixed synthesis rate of p_2 from 1 to 1000 nM/hr and the y-axes are the association constants K_a from 0.001 to 1 nM^{-1}, with both axes being log-scaled. For heterodimer without cooperative stability, the heatmap of the dimer abundance response to a 150% synthesis rate is plotted in (A). For heterodimer with cooperative stability, heatmaps of dimer abundance responses to a 150% synthesis rate are plotted for differing extents of cooperative stability, i.e., $\lambda_{\mathrm{momomer}}/\lambda_{\mathrm{dimer}}$=5 (B) and $\lambda_{\mathrm{momomer}}/\lambda_{\mathrm{dimer}}$=10 (C).

(D-F) Regime of upward controllability and an appropriate dimer/monomer ratio in the parameter space for the heterodimer formation system without ($\lambda_{\mathrm{momomer}}/\lambda_{\mathrm{dimer}}$=1) (D) or with ($\lambda_{\mathrm{momomer}}/\lambda_{\mathrm{dimer}}$>1) (E and F) cooperative stability. Upward controllability is defined as >120% dimer increase in response to a 150% synthesis rate increase. An appropriate dimer/monomer ratio is defined as >1.

Fig. S8. Downward controllability is comparable between systems with or without cooperative stability.

(A-B) Heatmaps of dimer abundance when the synthesis rate decreases by 50% within the physiological range, as described in Fig. 4, without (A) or with (B) cooperative stability. Dimer abundance is a percentage of the dimer abundance when the p_1 and p_2 synthesis rates are the same. (C-D) Regime in the parameter space for a heterodimer without (C) or with (D) cooperative stability and with a tolerance range>0 and at least 57% remaining when synthesis rate is decreased by 50%.

File S1. Derivation of the maximum tolerance range of protein complex formation systems with or without cooperative stability.

File S2. Source codes of the programs for model simulations and analysis.

Supplementary Text S1

In Supplementary Text S1 we derive the maximum tolerance score of protein complex formation systems with or without cooperative stability.

Mathematical deduction of the maximum tolerance score of a protein complex formation system lacking cooperative stability

The upper limit of the tolerance score is represented by the intersection, i.e., where the amounts of two protein species are the same, of the p_1 and p_3 response curves to C_1 (p_1 synthesis rate) variations:

$$p_1 = p_3 \qquad (1)$$

We solved equation (1) for C_1 by using the solutions of p_1 and p_3 at steady-state, as described under "Heterodimer" (see Materials and Methods) to obtain the upper limit:

$$upper\ limit = \frac{C_2\lambda_3}{\lambda_3} + C_2 - \frac{\lambda_1\lambda_2}{K_a\lambda_3} - \frac{\lambda_2}{K_a} \qquad (2)$$

Likewise, we obtained the lower limit by solving C_1 at the intersection of the p_2 and p_3 response curves:

$$lower\ limit = \frac{K_aC_2\lambda_3 + \lambda_1\lambda_2 + \lambda_2\lambda_3}{K_a(\lambda_2+\lambda_3)} \qquad (3)$$

Thus, $\frac{upper\ limit}{lower\ limit}$ can be written as:

$$\frac{upper\ limit}{lower\ limit} = \frac{(\lambda_2+\lambda_3)(K_aC_2\lambda_3 + K_aC_2\lambda_1 - \lambda_1\lambda_2 - \lambda_2\lambda_3)}{\lambda_3(K_aC_2\lambda_3 + \lambda_1\lambda_2 + \lambda_2\lambda_3)} \qquad (4)$$

For a protein complex lacking cooperative stability, the degradation rates of p_1, p_2, and p_3 are the same:

$$\lambda_1 = \lambda_2 = \lambda_3 = L \qquad (5)$$

Accordingly, we replaced all λ_i in equation (4) with a constant L to simplify the equation to:

$$\frac{upper\ limit}{lower\ limit} = \frac{(\mathrm{L}+\mathrm{L})\left(K_a C_2 \mathrm{L}+K_a C_2 \mathrm{L}-\mathrm{L}^2-\mathrm{L}^2\right)}{\mathrm{L}(K_a C_2 \mathrm{L}+\mathrm{L}^2+\mathrm{L}^2)}$$

$$= 4\,\frac{\left(K_a C_2 \mathrm{L}-\mathrm{L}^2\right)}{\left(K_a C_2 \mathrm{L}+2\mathrm{L}^2\right)} \qquad (6)$$

Multiplying both the numerator and denominator of equation (6) by $\frac{1}{K_a L}$, we get:

$$\frac{upper\ limit}{lower\ limit} = 4\,\frac{\left(C_2-\frac{\mathrm{L}}{K_a}\right)}{\left(C_2+\frac{2\mathrm{L}}{K_a}\right)} \qquad (7)$$

Since all parameters are greater than zero:

$$\left(C_2 - \frac{\mathrm{L}}{K_a}\right) < \left(C_2 + \frac{2\mathrm{L}}{K_a}\right)$$

$$\frac{\left(C_2-\frac{\mathrm{L}}{K_a}\right)}{\left(C_2+\frac{2\mathrm{L}}{K_a}\right)} < 1 \qquad (8)$$

Thus, the tolerance score cannot exceed 2 without cooperative stability:

$$\text{Tolerance score} = log_2\left[4\,\frac{\left(C_2-\frac{\mathrm{L}}{K_a}\right)}{\left(C_2+\frac{2\mathrm{L}}{K_a}\right)}\right] < \; log_2(4\cdot 1) = 2$$

Enhancing either K_a or C_2 can increase the tolerance score to reach the maximum value:

$$\lim_{K_a\to\infty} \frac{\left(C_2 - \frac{\mathrm{L}}{K_a}\right)}{\left(C_2 + \frac{2\mathrm{L}}{K_a}\right)} \sim 1$$

$$\lim_{C_2\to\infty} \frac{\left(C_2 - \frac{\mathrm{L}}{K_a}\right)}{\left(C_2 + \frac{2\mathrm{L}}{K_a}\right)} \sim 1$$

Moreover, if the upper limit is less than the lower limit, the tolerance score would be negative. Accordingly, we set negative tolerance scores as zero because monomers are more abundant than dimers even with stoichiometrically balanced inputs. We

further explored the condition for a protein complex to be formed as being more likely than for unassembled subunits to accumulate, at least within a certain range of synthesis rates:

$$\text{Tolerance score} = log_2\left[4\frac{\left(C_2-\frac{\text{L}}{K_a}\right)}{\left(C_2+\frac{2\text{L}}{K_a}\right)}\right] > 0, \quad 4\frac{\left(C_2-\frac{\text{L}}{K_a}\right)}{\left(C_2+\frac{2\text{L}}{K_a}\right)} > 1$$

$$4C_2 - \frac{4\text{L}}{K_a} > C_2 + \frac{2\text{L}}{K_a}$$

$$3C_2 > \frac{6\text{L}}{K_a}$$

Hence, we obtained the condition for a protein complex formation system lacking cooperative stability to achieve a dimer:monomer ratio >1:

$$C_2 > \frac{2\text{L}}{K_a} \tag{9}$$

Mathematical deduction of the maximum tolerance score of a protein complex formation system with cooperative stability

In Fig. S5, we demonstrate that the increase in tolerance score due to cooperative stability has a maximal value, and this value depends on the association constant and protein synthesis rate. In this section, we provide a mathematical deduction to describe the relationship between these model parameters and the maximum value of the tolerance score.

With cooperative stability, the degradation rate of the monomer is β-fold that of the dimer (i.e., monomers are degraded more efficiently), where $\beta > 1$:

$$\frac{\lambda_{monomer}}{\lambda_{dimer}} = \beta \tag{10}$$

Let the degradation rate of the dimer be a constant L:

$$\lambda_3 = L, \qquad \lambda_1 = \lambda_2 = \beta L.$$

By replacing λ_i in equation (4) with L and βL, the tolerance score of a protein complex with cooperative stability is:

$$Tolerance\ score = log_2 \frac{(\beta L + L)(K_a C_2 \beta L + K_a C_2 L - \beta^2 L^2 - \beta L^2)}{L(K_a C_2 L + \beta^2 L^2 + \beta L^2)}$$

Let τ be a function of β:

$$\tau\,(\beta) = \frac{(\beta L + L)(K_a C_2 \beta L + K_a C_2 L - \beta^2 L^2 - \beta L^2)}{L(K_a C_2 L + \beta^2 L^2 + \beta L^2)}$$

The derivative of $\tau\,(\beta)$ is:

$$\tau'(\beta) = \frac{-L(2\beta+1)\left(-\beta^3 L + \beta^2(K_a C_2 - 2L) + \beta(2K_a C_2 - L) + K_a C_2\right) + (\beta^2 L + \beta L + K_a C_2)(-3\beta^2 L + 2\beta(K_a C_2 - 2L) + 2K_a C_2 - L)}{(\beta L^2 + \beta L + K_a C_2)^2}$$

By solving the equation $\tau'(\beta) = 0$, we can determine the extrema of $\tau\,(\beta)$.

Hence, there is a maximum tolerance score for a protein complex with cooperative stability when β is:

$$\beta = \frac{4K_a C_2}{L \cdot \omega} - \frac{1}{3}(\omega + \omega^{-1} + 1) \qquad (11)$$

where ω is:

$$\omega = \sqrt[3]{-\frac{27{K_a}^2 C_2^2}{L^2} + \frac{9K_a C_2}{L} + 1 + \sqrt[2]{-\frac{(-12K_a C_2 + L)^3}{L^3} + \frac{\left(-27{K_a}^2 C_2^2 + 9K_a L C_2 + L^2\right)^3}{L^4}}}$$
